# Molecular dynamics provides insight into how N251A and N251Y mutations in the active site of *Bacillus licheniformis* RN-01 levansucrase disrupt production of long-chain levan

**DOI:** 10.1371/journal.pone.0204915

**Published:** 2018-10-02

**Authors:** Thassanai Sitthiyotha, Rath Pichyangkura, Surasak Chunsrivirot

**Affiliations:** 1 Department of Biochemistry, Faculty of Science, Chulalongkorn University, Pathumwan, Bangkok, Thailand; 2 Structural and Computational Biology Research Group, Department of Biochemistry, Faculty of Science, Chulalongkorn University, Pathumwan, Bangkok, Thailand; UMR-S1134, INSERM, Université Paris Diderot, INTS, FRANCE

## Abstract

Produced by levansucrase, levan and levan oligosaccharides (GF_n_) have potential applications in food and pharmaceutical industries such as prebiotics, anti-tumor and anti-inflammatory agents. Previous study reported that *Bacillus licheniformis* RN-01 levansucrase could produce levan oligosaccharides and long-chain levan. However, its N251A and N251Y mutants could effectively produce short-chain oligosaccharides upto GF_3,_ but they could not produce long-chain levan. We hypothesized that these mutations probably reduced GF_3_ binding affinity in levansucrase active site that contains fructosyl-Asp93 intermediate and caused GF_3_ to be in an unfavorable orientation for transfructosylation; therefore, levansucrase could not effectively extend GF_3_ by one fructosyl residue to produce GF_4_ and subsequently long-chain levan. However, these mutations probably did not significantly reduce binding affinity or drastically change orientation of GF_2_; therefore, levansucrase could still extend GF_2_ to produce GF_3_. Using this hypothesis, we employed molecular dynamics to investigate effects of these mutations on GF_2_/GF_3_ binding in levansucrase active site. Our results reasonably support this hypothesis as N251A and N251Y mutations did not significantly reduce GF_2_ binding affinity, as calculated by MM-GBSA technique and hydrogen bond occupations, or drastically change orientation of GF_2_ in levansucrase active site, as measured by distance between atoms necessary for transfructosylation. However, these mutations drastically decreased GF_3_ binding affinity and caused GF_3_ to be in an unfavorable orientation for transfructosylation. Furthermore, the free energy decomposition and hydrogen bond occupation results suggest the importance of Arg255 in GF_2_/GF_3_ binding in levansucrase active site. This study provides important and novel insight into the effects of N251A and N251Y mutations on GF_2_/GF_3_ binding in levansucrase active site and how they may disrupt production of long-chain levan. This knowledge could be beneficial in designing levansucrase to efficiently produce levan oligosaccharides with desired length.

## Introduction

**Fig 1:**
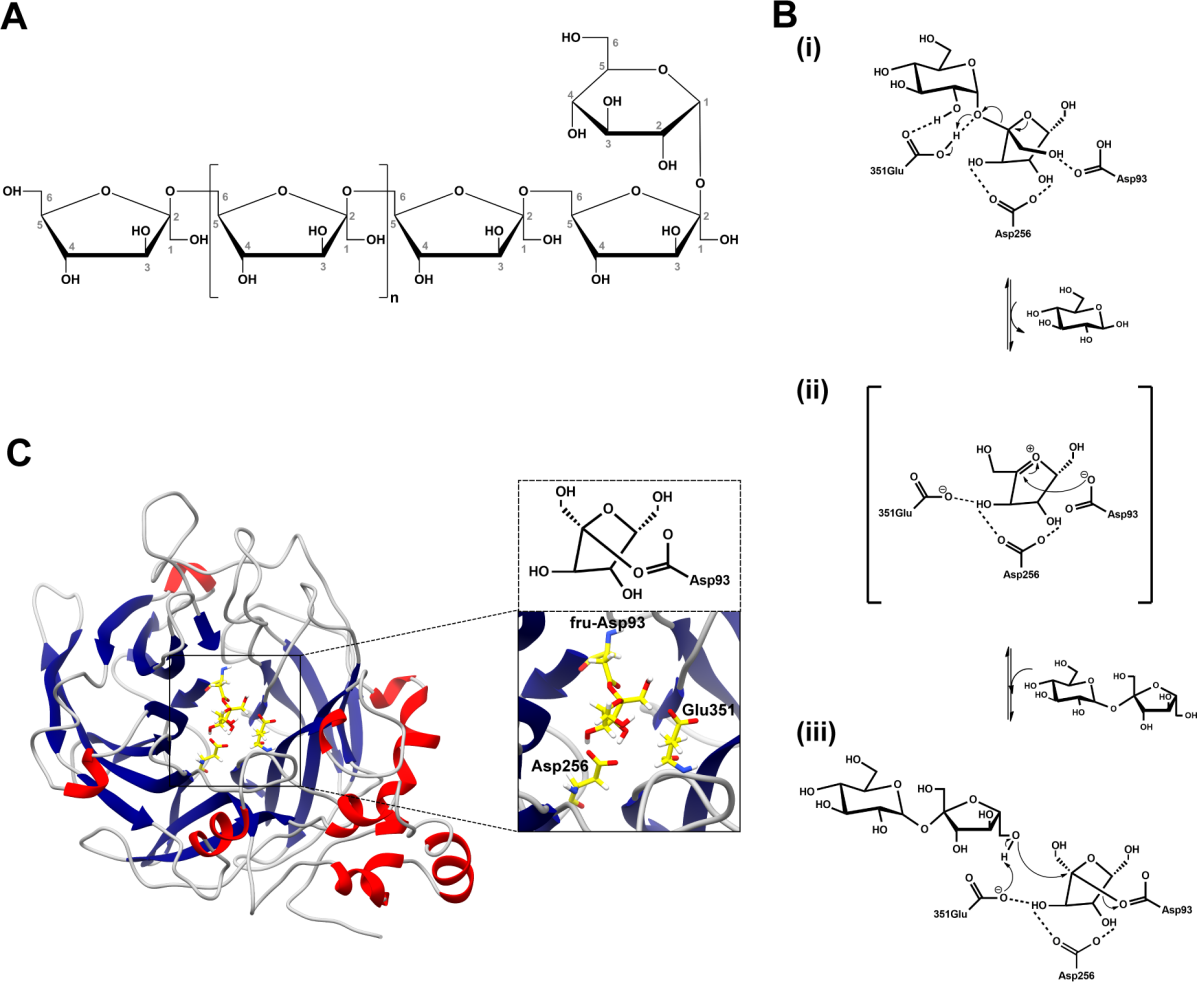
Levan, proposed reaction mechanism and levansucrase. (A) An example of levan structure with no branching. (B) Proposed reaction mechanism of levansucrase; (i) sucrose binds in the active site of levansucrase, (ii) the fructosyl-Asp93 intermediate is formed, while glucose is leaving the active site, (iii) sucrose binds at the acceptor binding site. The β-(2, 6) linkage is formed between sucrose and the fructosyl residue of the fructosyl-Asp93 intermediate to extend the levan chain. The bond between the fructosyl residue and Asp93 is broken, and the product is released. (C) Homology model of *Bacillus licheniformis* RN-01 levansucrase with the fructosyl-Asp93 intermediate.

Levan and levan oligosaccharides (GF_n_) are natural fructans that contain one terminal glucopyranosyl residue and D-fructofuranosyl repeating unit linked by β-(2, 6) linkage in a main chain with some possible branching points linked by β-(2, 1) linkages [1] (Fig 1A). Properties of levan and levan oligosaccharides depend on their lengths and branching degrees [2], and they have various beneficial properties such as high-water solubility [3] and low intrinsic viscosity [4] for food, cosmetics and pharmaceutical industries. In the food industry, levan and levan oligosaccharides can be used as a prebiotic ingredient [5], encapsulating agent, emulsifier, thickener [3] and cholesterol lowering agent [6]. They can also be used as a component in cosmetics to alleviate skin irritation and moisturize skin [7]. For pharmaceutical industry, they could potentially be used as anti-tumor, anti-inflammatory and anti-viral agents [8].

Levan and levan oligosaccharides are synthesized by levansucrase that is mainly found in various microorganism, including *Bacillus subtilis* [9], *Rahnella aquatilis* [10], *Zymomonas mobilis* [11], *Leuconostoc mesenteroides* [12] and *Leuconostoc citreum* [1]. The mechanism of levansucrase was proposed to occur in two steps (Fig 1B) [13]. In the first step, sucrose is bound in the active site, and the fructosyl residue is stabilized by the transition state stabilizer (Asp256). The acid-base catalyst, Glu351, acts as a general acid, protonating the glycosidic oxygen of sucrose. Glucose is released, and oxocabenium ion of the fructosyl residue is formed. Then, a nucleophile (Asp93) attacks C2 of the oxocabenium ion, and the covalent fructosyl-enzyme intermediate is formed. In the second step, sucrose binds in the acceptor binding site. Glu351 acts as a general base that removes a proton from O6 of the non-reducing end of the acceptor. Then, this O6 attacks the fructosyl C2 of the covalent fructosyl-enzyme intermediate, creating the β-(2, 6) linkage to extend the levan chain. The bond between the fructosyl residue and Asp93 is broken, and the product is released [13].

Previous experimental study by Nakapong reported that *Bacillus licheniformis* RN-01 levansucrase could produce levan oligosaccharides and long-chain levan. However, its N251A and N251Y mutants could effectively produce short-chain oligosaccharides upto GF_3_, but they could not produce long-chain levan at 323 K and pH 6 [14]. In other words, the wild type could extend GF_2_ and GF_3_ by one fructosyl residue to produce GF_3_ and GF_4,_ respectively, while the mutants could extend GF_2_ by one fructosyl residue to produce GF_3,_ but they could not effectively extend GF_3_ to produce GF_4_ and subsequently long-chain levan. However, the molecular-level understanding on how these mutations cause production of short-chain products is lacking.

Molecular dynamics (MD) simulations is widely used to elucidate conformational changes of enzymes over a period of time and to gain insights into the interactions between enzymes and substrates that may not be accessible by experiments [15–20]. This method can also be used to calculate the binding free energy of ligand binding in macromolecules [17, 21]. However, to our knowledge, MD technique has not been employed to investigate levan oligosaccharides binding in the active site of levansucrase and the effects of mutations on the binding of these substrates.

In this study, MD simulations were performed at experimental temperature and pH on six complexes: GF_2_-wild-type levansucrase (GF_2_-LS_wt_), GF_2_-N251A mutant levansucrase (GF_2_-LS_N251A_), GF_2_-N251Y mutant levansucrase (GF_2_-LS_N251Y_), GF_3_-wild-type levansucrase (GF_3_-LS_wt_), GF_3_-N251A mutant levansucrase (GF_3_-LS_N251A_) and GF_3_-N251Y mutant levansucrase (GF_3_-LS_N251Y_) to elucidate the effects of N251A and N251Y mutations on the binding of GF_2_/GF_3_ in the active site of *Bacillus licheniformis* RN-01 levansucrase that contains the fructosly-Asp93 intermediate (fru-Asp93). This molecular-level understanding on GF_2_/GF_3_ binding in *Bacillus licheniformis* RN-01 levansucrase might be beneficial for designing mutants that can produce levan oligosaccharides with desired lengths.

## Materials and methods

### Structure preparation

The structures of GF_2_ and GF_3_ were constructed using the LEaP module in AMBER14 [22] and the GLYCAM06j-1force field parameters [23]. To remove unfavorable interactions, these structures were minimized by 2,500 steps of steepest descent and 2,500 steps of conjugate gradient. The target sequence of *Bacillus licheniformis* RN-01 levansucrase (GenBank ID: ACI15886.1) was obtained from the National Center for Biotechnology Information (NCBI). SWISS-MODEL server [24–27] was used to construct the homology model of levansucrase from *Bacillus licheniformis* RN-01 based on the crystal structure of *Bacillus subtilis* levansucrase (PDB ID: 1OYG [28]), which has the highest sequence identity to the target sequence. The quality of the homology model was evaluated by Ramachandran plot produced by the RAMPAGE server [29]. S1 Fig shows that a majority of its residues are in favored region (96.0%) and allowed region (3.3%), indicating reasonable quality of this homology model. Moreover, the catalytic residues (Asp93, Asp256 and Glu351) of this homology model were found in positions, where they should be able to catalyze the transfructosyation (Fig 1C). All ionizable amino acids were protonated at pH = 6, using the H^++^ server [30]. To construct the structure of fru-Asp93, the initial structure of Asp86 and fructosyl residue were taken from the crystal structure of *Bacilus subtilis* levansucrase in complex with sucrose (PDB ID: 1PT2 [28]). GaussView05 program [31] was used to create a bond between OD2 of Asp and C2 of the fructosyl residue. The atomic charges and the electrostatic potential (ESP) charges of fru-Asp93 were calculated using the HF/6-31G* basis set in the Gaussian09 program [32]. Using Antechamber module in AMBER14, the ESP charges of fru-Asp93 intermediate was converted into restrained ESP (RESP) charges, and other force filed parameters of fru-Asp93 intermediate were generated from general AMBER force field (GAFF). The LEaP module was then used to construct the structure of levansucrase with fru-Asp93 in its active site, using ff14SB force field (Fig 1C).

### Identification of catalytically competent binding conformations and molecular dynamics

To determine whether Autodock vina [33] and its parameters were appropriate for the studied systems, the crystal sucrose was redocked into the active site of the crystal structure of *Bacillus subtilis* levansucrase (1PT2). The best docked and crystal binding conformations were compared and found to be reasonably similar with the RMSD value of 0.64 Å (S2 Fig), indicating that Autodock Vina and its parameters were appropriate for this system. To determine catalytically competent binding conformations, Autodock Vina was employed to dock GF_2_/GF_3_ in the active site of the homology model of wild-type levansucrase with fru-Asp93 to create GF_2_-LS_wt_ and GF_3_-LS_wt_ complexes. A grid box of 40 Å x 40 Å x 40 Å with a grid spacing of 1 Å was employed. 20 independent docking runs were performed for each ligand, where each run gave nine possible binding conformations. In order for the wild type to be able to extend the levan chain, GF_2_/GF_3_ should bind in catalytically competent orientations, where O6 of the non-reducing end of GF_2_/GF_3_ turns toward C2 of the fructosyl residue of fru-Asp93. Employing this assumption, only binding conformations that have O6 of the non-reducing end of GF_2_/GF_3_ turns toward C2 of the fructosyl residue of fru-Asp93 were selected. The binding conformations that passed this criterion were later clustered by MMTSB tool set [34] based on their structural similarities as measured by the RMSD values of heavy atoms. To identify a reasonable representative binding conformation of each cluster, a binding conformation that is most similar to the average structure of all members of each cluster was chosen to be a centroid. The centroid of each cluster was immersed in an isomeric truncated octahedral box of TIP3P water molecules with the buffer distance of 13 Å using the LEaP module. Chloride ions (Cl^-^) were added to neutralize all systems. To reduce unfavorable interactions, the complexes were minimized with the five step procedure. All steps include 5,000 steps of steepest descent and 5,000 steps of conjugate gradient with different restraints on the proteins. Initially, to relax each system, the hydrogen atoms and water molecules were minimized, while heavy atoms of protein were restrained with a force constant of 5 kcal/ (mol Å^2^). The backbone of the protein was subsequently restrained with force constants of 10, 5 and 1 kcal/ (mol Å^2^), respectively. Finally, the entire system was minimized without any restraining force. The GPU (CUDA) version of PMEMD module of the AMBER14 was employed to simulate all systems under the periodic boundary condition [35–37]. The SHAKE algorithm [38] was used to constrain all bonds involving hydrogen atoms, allowing a simulation time step of 0.002 ps. A cutoff distance of 12 Å was used for non-bonded interactions, and the particle mesh Ewald method was applied to calculate the long-range electrostatic interaction [38]. The Langevin dynamics technique [39] was employed to control the temperature with a collision frequency of 1.0 ps^-1^. All systems were heated from 0 K to the experimental temperature of 323 K (50°C) for 200 ps in the NVT ensemble, while the backbone of proteins were restrained with a force constant of 10 kcal/ (mol Å^2^). Subsequently, all systems were equilibrated for 300 ps with no restraint in the NVT ensemble. These systems were further simulated for 80 ns in the NPT ensemble at 323 K and 1 atm. With the assumption that catalytically competent binding conformations should have the position of O6 of the non-reducing end of GF_2_/GF_3_ that is not too far from that of C2 of the fructosyl residue of fru-Asp93 after simulations, the distances between O6 of the non-reducing end of GF_2_/GF_3_ and C2 of the fructosyl residue of fru-Asp93 (O6-C2 distance) of all centroids were measured. The centroids with the O6-C2 distances greater than 5 Å were eliminated. One centroid of GF_2_ binding conformations and one centroid of GF_3_ binding conformations passed this criterion. Since it may still be possible that the selected centroid may not necessary be the most stable binding conformation of the cluster, similar setup, minimization and MD procedure were also performed on two additional binding conformations of GF_2_/GF_3_ that are in the same cluster as the selected centroid. These binding conformations were second and third most similar to the average structure of all members of each cluster. With the assumption that catalytically competent binding conformations of GF_2_/GF_3_ in the active site of the wild type should be the ones, where GF_2_/GF_3_ stably binds in its active site, the binding conformations, whose heavy-atom RMSD of GF_2_/GF_3_ during 60–80 ns (the last 20 ns of the simulation) have the lowest values and fluctuation out of the three binding conformations, were chosen to be the catalytically competent binding conformations of the wild type complex (GF_2_-LS_wt_ and GF_3_-LS_wt_ complexes). To construct the structures of the mutant complexes, Asn251 of the selected binding conformations of GF_2_-LS_wt_ and GF_3_-LS_wt_ complexes were mutated to Ala251 to build GF_2_-LS_N251A_ and GF_3_-LS_N251A_ complexes, and it was mutated to Tyr251 to build GF_2_-LS_N251Y_ and GF_3_-LS_N251Y_ complexes. Similar setup, minimization and MD were performed on the mutant systems.

In terms of analyses, the RMSD values with respect to the minimized structure were calculated to monitor the stability of all systems. Since the RMSD values of all systems were stable around 60–80 ns, these trajectories were used for further analyses. To measure the proximity between atoms necessary for transfructosylation, the O6-C2 distances of all systems were measured. To measure binding affinity between GF_2_/GF_3_ and levansucrase, total binding free energies and decomposition of free energies per residue were calculated by Molecular Mechanics/Generalized Born Surface Area (MM/GBSA) method. MM/GBSA technique [40, 41] is widely employed to approximate the binding affinities, as calculated by binding free energies, of small ligands to macromolecules [42]. This method is stable, reproducible [42] and giving promising results in correctly ranking the molecules with known affinity to their target proteins [43–50]. This technique was also employed for rigorous free energy decomposition into contributions from different groups of atoms or types of interaction in various studies to determine important binding residues [51–54].

Hydrogen bond interactions between GF_2_/GF_3_ and levansucrase were determined by calculating hydrogen bond occupations between amino acid residues and GF_2_/GF_3_. In this study, a hydrogen bond occurred if the following criteria were met: (i) a proton donor-acceptor distance ≤ 3.5 Å and (ii) a donor-H-acceptor bond angle ≥ 120°. Strong and medium hydrogen bonds were defined as hydrogen bonds with occupation > 75% and 50–75%, respectively. Weak hydrogen bonds were defined as hydrogen bonds with occupation < 50% but ≥ 25%.

## Results and discussion

### System stability

**Fig 2:**
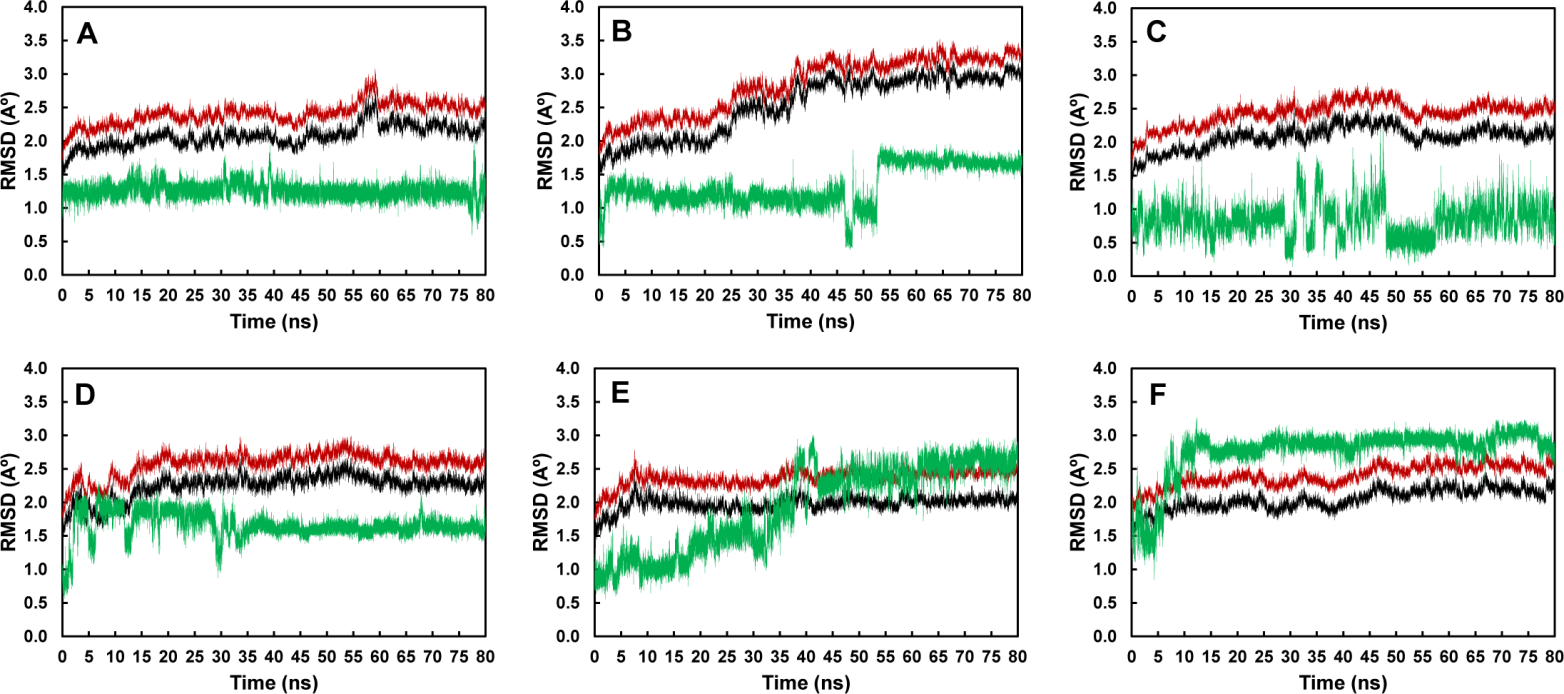
RMSD plots of A) GF_2_-LS_wt_, B) GF_2_-LS_N251A_, C) GF_2_-LS_N251Y_, D) GF_3_-LS_wt_, E) GF_3_-LS_N251A_ and F) GF_3_-LS_N251Y_ complexes. The RMSD values of all atoms, backbone atoms and ligand atoms are shown in red, black and green, respectively.

Using the minimized structures as references, the RMSD values of all atoms, backbone atoms and ligand atoms of all systems were calculated to determine the stabilities of these systems and identify appropriate trajectories for further analyses (Fig 2). As shown by these plots, the simulations of all systems were likely to reach equilibrium around 80 ns. As a result, the 60–80 ns trajectories of all systems were employed for further analyses.

### The proximity between atoms necessary for transfructosylation

**Fig 3:**
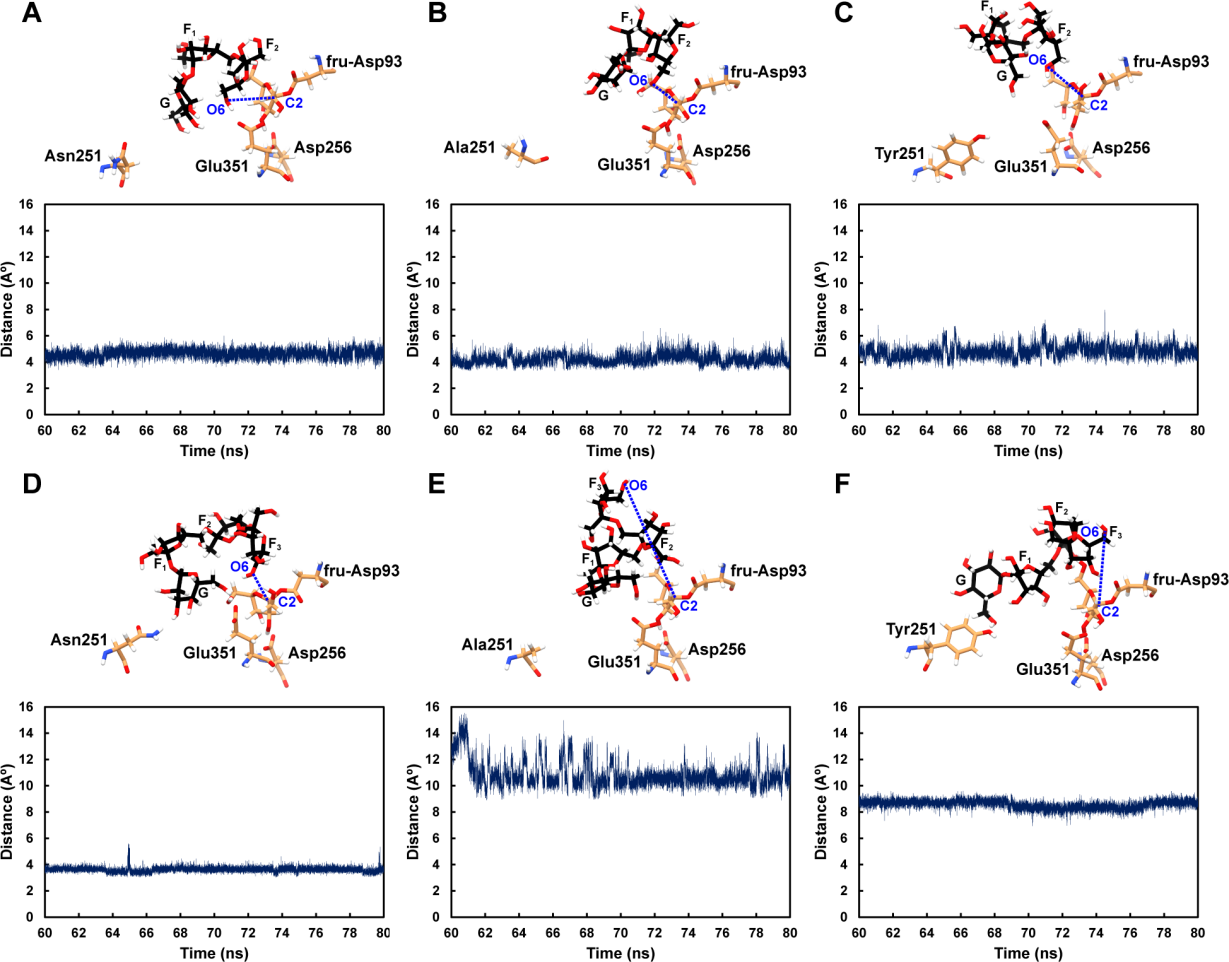
The distance between O6 of the non-reducing end of GF_2_/GF_3_ and C2 of the fructosyl residue of fru-Asp93: A) GF_2_-LS_wt_, B) GF_2_-LS_N251A_, C) GF_2_-LS_N251Y_, D) GF_3_-LS_wt_, E) GF_3_-LS_N251A_ and F) GF_3_-LS_N251Y_ complexes.

With the assumption that the system that allow transfructosylation to occur should be the one that has O6 of the non-reducing end of GF_2_/GF_3_ turning toward C2 of the fructosyl residue of fru-Asp93, and these two atoms should not be too far from each other, the O6-C2 distances of all systems were measured as shown in Fig 3 and S3 Fig. The O6-C2 distances of GF_2_-LS_wt_, GF_2_-LS_N251A_ and GF_2_-LS_N251Y_ are reasonable and quite stable during the 60–80 ns simulations. These findings suggest that transfructosylation should be able to occur in these systems, i.e., the wild type, the N251A and N251Y mutants should be able to extend GF_2_ by one fructosyl residue to create GF_3_. These results support the previous experimental findings that the wild type, the N251A and N251Y mutants could produce GF_3_.

**Fig 4:**
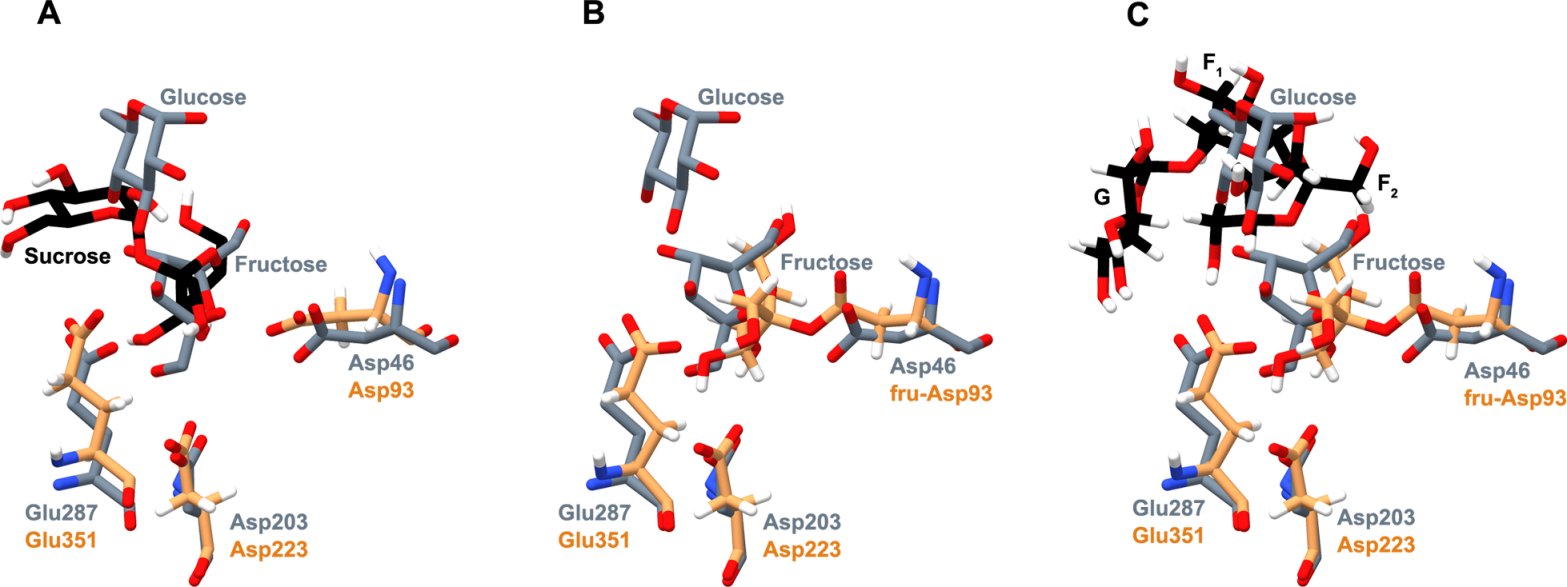
Superimpositions between the crystal structure of *Erwinia amylovora* in complex with fructose and glucose (grey) and (A) the homology model of *Bacillus licheniformis* RN-01 levansucrase (orange) with docked sucrose (black), (B) the homology model of *Bacillus licheniformis* RN-01 levansucrase containing fru-Asp93 intermediate (orange) and (C) the homology model of *Bacillus licheniformis* RN-01 levansucrase containing fru-Asp93 intermediate (orange) with catalytically competent binding conformation of GF_2_ (black).

Superimpositions between the crystal structure of *Erwinia amylovora* levansucrase in complex with fructose and glucose (PDB ID: 4D47 [55]) and the homology model of *Bacillus licheniformis* RN-01 levansucrase with docked sucrose, between 4D47 and the homology model of *Bacillus licheniformis* RN-01 levansucrase containing fru-Asp93 intermediate as well as between 4D47 and the homology model of *Bacillus licheniformis* RN-01 levansucrase containing fru-Asp93 intermediate with catalytically competent binding conformation of GF_2_ are shown Fig 4. These results show that the fructosyl reside of sucrose and fructosyl residue of fru-Asp93 are in similar position to that of fructose in the crystal structure of *Erwinia amylovora* levansucrase (Fig 4A and 4B). Their orientations are slightly different probably because fructose in the crystal structure of *Erwinia amylovora* levansucrase is the hydrolysis product of sucrose and it does not connect to other residue; therefore, it has more flexibility in terms of orientation than the fructosyl residue of sucrose/fru-Asp93. Moreover, Fig 4C shows that the position of the fructosyl residue of the non-reducing end of GF_2_ is close to that of glucose in the crystal structure of *Erwinia amylovora* levansucrase.

For the systems involving GF_3_, the O6-C2 distance of GF_3_-LS_wt_ is reasonable and stable, suggesting that transfructosylation should be able to occur. However, N251A and N251Y mutations drastically increase the O6-C2 distances to around 10.8 Å and 8.6 Å for GF_3_-LS_N251A_ and GF_3_-LS_N251Y_ complexes, respectively. For these two mutant complexes, O6 of the non-reducing end of GF_3_ is too far from C2 of the fructosyl residue of fru-Asp93 for transfructosylation to occur. Moreover, O6 of the non-reducing end of GF_3_ also points away from C2 of the fructosyl residue of fru-Asp93. Therefore, the orientations of GF_3_ in these two mutant systems are not favorable for transfructosylation to occur, and these mutants should not be able to effectively extend GF_3_ by one fructosyl residue to produce GF_4_. These findings support the previous experimental results that the wild type could produce GF_4_, while the N251A and N251Y mutants could not effectively produce GF_4_ and long-chain levan.

### Binding free energies

To determine if binding affinity is an important factor associated with the experimental results that the wild type, N251A and N251Y mutants could extend GF_2_ to produce GF_3_, while only the wild type could effectively extend GF_3_ to produce GF_4_, MM-GBSA method was employed to calculate the binding free energies of GF_2_-LS_WT_, GF_2_-LS_N251A_, GF_2_-LS_N251Y_, GF_3_-LS_wt_, GF_3_-LS_N251A_ and GF_3_-LS_N251Y_ complexes during the 60–80 ns trajectories (Table 1). The binding free energies of GF_2_-LS_wt_, GF_2_-LS_N251A_ and GF_2_-LS_N251Y_ complexes are -4.7±0.9, -10.1±0.8 and -4.5±0.8 kcal/mol, respectively. These results suggest that N251A and N251Y mutations did not significantly reduce the binding affinities of GF_2_ in the active site of levansucrase. Since the distances between atoms necessary for transfructosylation of these systems are also reasonable, the wild type, N251A and N251Y mutants should all be able to bind GF_2_, extend it by one fructosyl residue and produce GF_3_, supporting the experimental results. In terms of GF_3_ binding, the binding free energies of GF_3_-LS_WT_, GF_3_-LS_N251A_ and GF_3_-LS_N251Y_ complexes are -20.5±0.7, 1.1±0.9 and -8.7±0.8 kcal/mol, respectively. These results show that the binding affinities of GF_3_-LS_N251A_ and GF_3_-LS_N251Y_ complexes are worse than that of the GF_3_-LS_WT_ complex, suggesting that these mutations reduce the binding affinities of GF_3_ in the active site of the mutants as compared to that of the wild type. Since the distance between atoms necessary for transfructosylation is reasonable only for the wild-type complex, these results suggest that only the wild type could potentially bind GF_3_, extend it by one fructosyl residue and produce GF_4_, while the N251A and N251Y mutants could not tightly bind GF_3_ to effectively produce GF_4_, supporting the experimental results.

**Table 1:** The Binding free energies (kcal/mol) and their components of GF_2_-LS_wt_, GF_2_-LS_N251A_, GF_2_-LS_N251Y_, GF_3_-LS_wt_, GF_3_-LS_N251A_, and GF_3_-LS_N251Y_ complexes.

System	ΔE_vdW_	ΔE_ele_	ΔG_pol_	ΔG_np_	^a)^ΔG_solv_	-TΔS_tot_	^b)^ΔG_bind_	Standard error of the mean of ΔG_bind_
**GF**_**2**_**-LS**_**wt**_	-35.3	-55.9	66.4	-5.6	60.8	25.7	-4.7	0.9
**GF**_**2**_**-LS**_**N251A**_	-38.2	-66.0	71.8	-5.7	66.1	28.0	-10.1	0.8
**GF**_**2**_**-LS**_**N251Y**_	-38.0	-47.8	60.3	-5.6	54.7	26.5	-4.5	0.8
**GF**_**3**_**-LS**_**wt**_	-47.4	-103.3	101.1	-7.9	93.2	37.0	-20.5	0.7
**GF**_**3**_**-LS**_**N251A**_	-45.7	-36.3	62.6	-6.2	56.4	26.7	1.1	0.9
**GF**_**3**_**-LS**_**N251Y**_	-54.7	-50.3	74.7	-7.4	67.3	29.0	-8.7	0.8

a) ΔG_solv_ = ΔG_pol_ + ΔG_np_

b) ΔG_bind_ = ΔE_vdW_ + ΔE_ele_ + ΔG_solv_—TΔS_tot_

**Fig 5:**
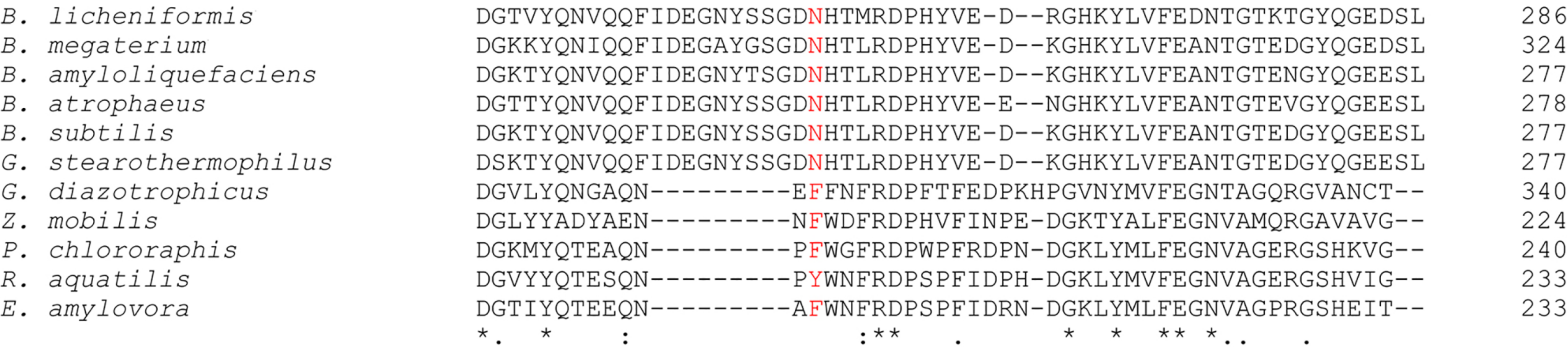
The multiple sequence alignment of the sequence near Asn251 of *B*. *licheniformis* RN-01 levanscurase and levansucrase from Gram-positive bacteria such as *B*. *megaterium* [56], *B*. *amyloliquefaciens* [57], *B*. *atrophaeus* [58], *B*. *subtilis* [9], and *B*. *stearothermophilus* [59], and from Gram-negative bacteria such as *G*. *diazotrophicus* [60], *Z*. *mobilis* [11], *P*. *chlororaphis* [61], *R*. *aquatilis* [62] and *E*. *amylovora* [63].

Levansucrase from Gram-positive bacteria generally produce long-chain levan polymer, while that from Gram-negative bacteria produce short-chain levan oligosaccharides [55]. *Bacillus licheniformis* RN-01 levansucrase is from Gram-positive bacteria; therefore, it usually produces long-chain levan polymer. However, its N251A and N251Y mutants could effectively produce short-chain oligosaccharides, and they could not produce long-chain levan. The multiple sequence alignment of *B*. *licheniformis* RN-01 levanscurase and levansucrase from Gram-positive bacteria such as *B*. *megaterium* [56], *B*. *amyloliquefaciens* [57], *B*. *atrophaeus* [58], *B*. *subtilis* [9], and *B*. *stearothermophilus* [59], and from Gram-negative bacteria such as *G*. *diazotrophicus* [60], *Z*. *mobilis* [11], *P*. *chlororaphis* [61], *R*. *aquatilis* [62] and *E*. *amylovora* [63] shows that Asn251 of levansucrase from Gram-positive bacteria is generally conserved, while that from Gram-negative bacteria is mutated to other residues such as Phe or Tyr (Fig 5 and S4 Fig). These mutations could potentially reduce the binding affinity to substrates such as GF_3_ in the active site of levansucrase from Gram-negative bacteria and could potentially disrupt the production of long-chain levan polymer, similar to the N251A and N251Y mutations of *Bacillus licheniformis* RN-01 levansucrase.

**Fig 6:**
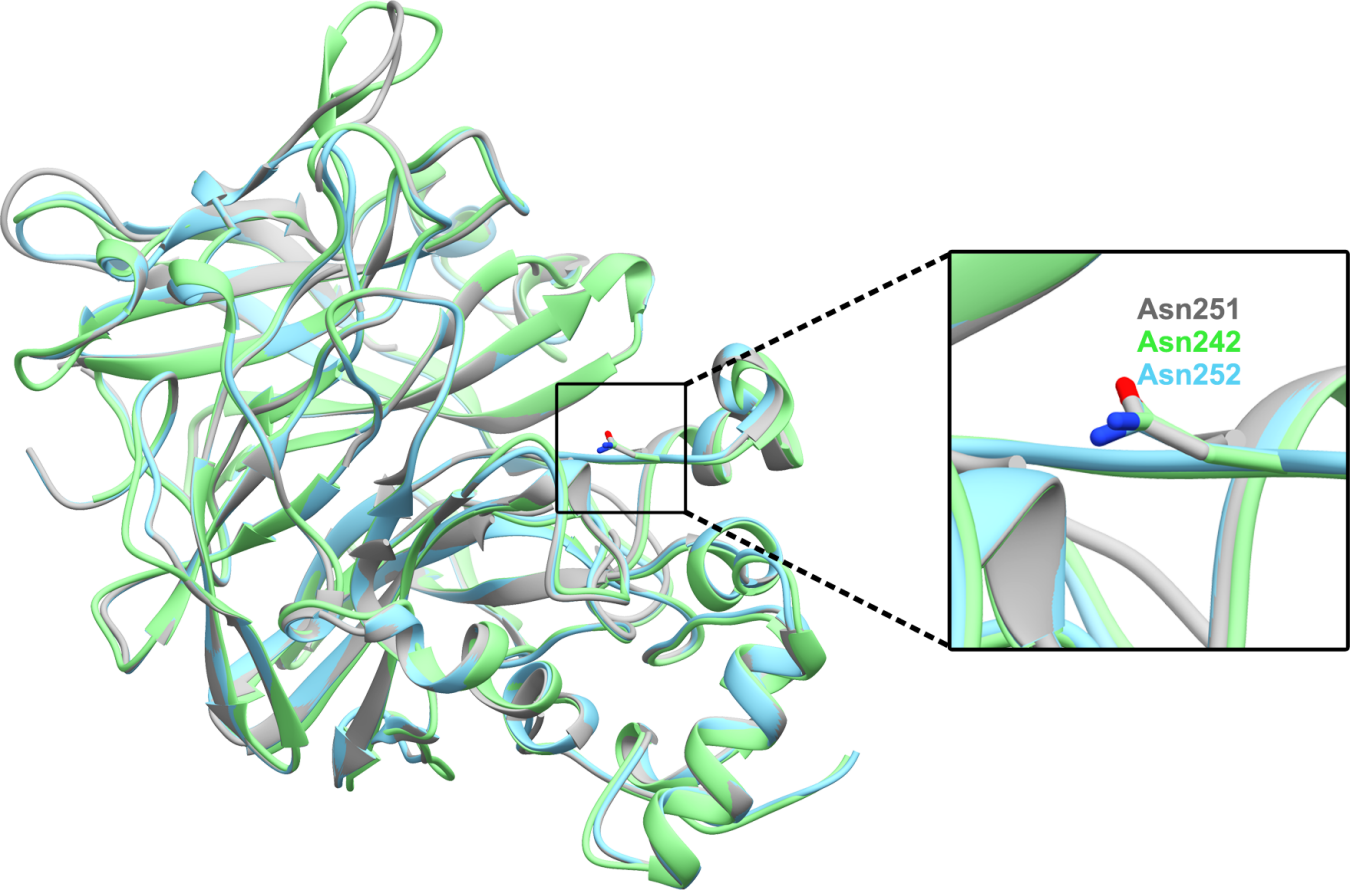
The superimposition of the homology model of *Bacillus licheniformis* RN-01 levansucrase (grey) with most similar levansucrase structures from *B*. *sutilis* (PDB ID: 1OYG [28], green) and *B*. *megaterium* (PDB ID: 3OM2 [65], blue) from PDBeFold.

Moreover, PDBeFold [64] was employed to perform structural superposition of our model with available crystal structures. S1 Table shows crystal structures of enzymes that are most similar to our model. Fig 6 shows the superimposition of our model with most similar levansucrase structures from *B*. *sutilis* (PDB ID: 1OYG [28]) and *B*. *megaterium* (PDB ID: 3OM2 [65]). These results show that the overall structure of our model is similar to the crystal structures of levansucrase from *B*. *sutilis* and *B*. *megaterium*. Additionally, the positions and orientations of N251 of our model and these most similar structures are very similar.

In terms of binding free energy components of GF_2_ binding, the main components contributing to the substrate binding affinities of GF_2_-LS_wt_, GF_2_-LS_N251A_, GF_2_-LS_N251Y_ complexes are the electrostatic interaction terms (ΔE_ele_) as they have the most favorable values that are in the range of -66.0 –-47.8 kcal/mol. Other terms favor GF_2_ binding are the van der Waals energy terms (ΔE_vdw_), which are in the range of -38.2 –-35.3 kcal/mol, and the non-polar solvation terms (ΔG_np_), which are in the range of -5.7 –-5.6 kcal/mol. The polar solvation terms (ΔG_pol_) have unfavorable contribution to GF_2_ binding, and they are in the range of 60.3–71.8 kcal/mol.

In terms of GF_3_ binding, the main component contributing to the substrate binding affinity of GF_3_-LS_wt_ complex is ΔE_ele_ with the value of -103.3 kcal/mol. ΔE_vdw_ and ΔG_np_ are also favorable with the values of -47.4 and -7.9 kcal/mol, respectively. ΔG_pol_ is unfavorable with the value of 101.1 kcal/mol. However, the main component contributing to GF_3_ binding affinities of GF_3_-LS_N251A_ and GF_3_-LS_N251Y_ complexes are ΔE_vdw_ with the values of -45.7 and -54.7 kcal/mol, respectively. The values of ΔE_ele_ of GF_3_-LS_N251A_ (-36.3 kcal/mol) and GF_3_-LS_N251Y_ (-50.3 kcal/mol) complexes are significantly worse than that of GF_3_-LS_wt_ complex (-103.3 kcal/mol). These results were probably caused by the fact that GF_3_ could not bind in a favorable orientation in the active sites of the N251A and N251Y mutants. In this case, GF_3_ could not form as many favorable interactions with residues in the active sites of the mutants as with those of the wild type. Additionally, ΔG_np_ of GF_3_-LS_N251A_ and GF_3_-LS_N251Y_ are favorable with the values of -6.2 and -7.4 kcal/mol. Their ΔG_pol_ values are unfavorable with the values of 62.6 and 74.7 kcal/mol.

### Per residue substrate-enzyme interactions

**Fig 7:**
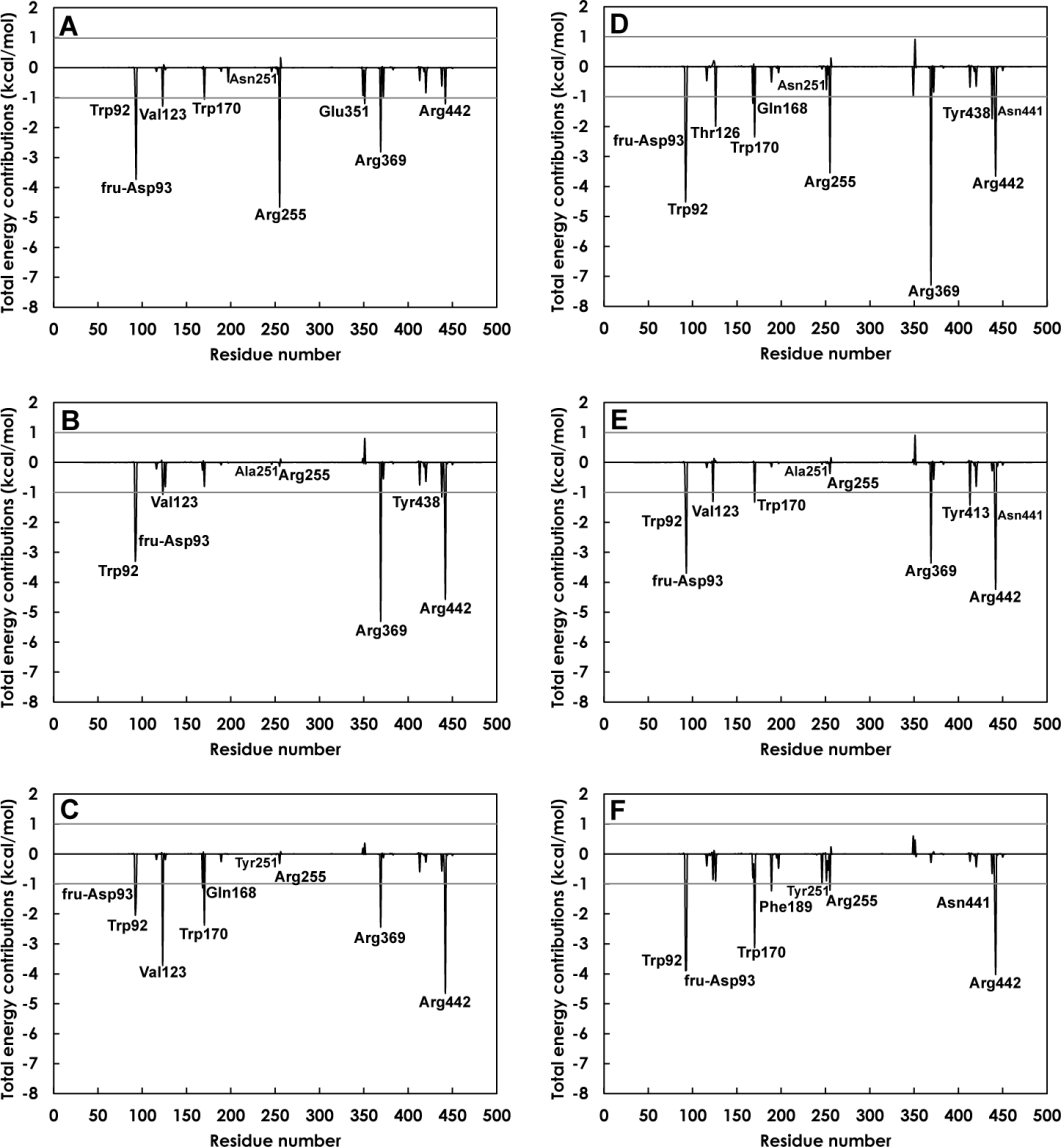
Per-residue decomposition of binding free energy contributions of A) GF_2_-LS_wt_, B) GF_2_-LS_N251A_, C) GF_2_-LS_N251Y_, D) GF_3_-LS_wt_, E) GF_3_-LS_N251A_ and F) GF_3_-LS_N251Y_ complexes.

To identify important binding residues that make major contributions to the calculated binding free energies as well as the effects of N251A and N251Y mutations on the binding residues, the values of free energy decomposition on a per residue basis (${\Delta G}_{bind}^{residue}$) were calculated as shown in Fig 7. In this study, an importance binding residue was defined to be a residue with the total energy contribution better than -1.0 kcal/mol. For GF_2_-LS complexes, residues with energy contribution better than -1 kcal/mol for all three complexes are Trp92, fru-Asp93, Val123, Arg369 and Arg442, indicating their importance in GF_2_ binding in the active sites of wild-type and mutant levansucrase. However, there are also residues with total energy contribution better than -1 kcal/mol in the wild-type complex, but not in the mutant complexes such as Trp170, Arg255 and Glu351, suggesting their importance in GF_2_ binding only in the active site of wild-type levansucrase. For GF_3_-Ls complexes, Trp92, fru-Asp93, Trp170, Asn441 and Arg442 have energy contribution better than -1 kcal/mol for all three systems, suggesting their importance in GF_3_ binding in the active sites of wild-type and mutant levansucrase. Thr126, Gln168, Arg255, Arg369 and Tyr438 have energy contribution better than -1 kcal/mol in the wild-type complex but not in the mutant complexes, suggesting their importance in GF_3_ binding only in the active site of wild-type levansucrase.

In terms of the mutated residue 251, we found that the N251A and N251Y mutations did not cause significant changes to the total energy contribution of residue 251. However, these mutations caused significant changes to the total energy contributions of other residues, especially Arg255. For GF_2_-LS complexes, the value of the total energy contribution of Arg255 was changed from -4.7 kcal/mol in the wild-type complex to -0.1 kcal/mol in the N251A mutant complex and to -0.3 kcal/mol in the N251Y mutant complex. For GF_3_-LS complexes, the value of the total energy contribution of Arg255 was changed from -3.5 kcal/mol in the wild-type complex to -0.4 kcal/mol in the N251A mutant complex and to -1.2 kcal/mol in the N251Y mutant complex. These results suggest the importance of Arg255 in GF_2_/ GF_3_ binding.

### Hydrogen bond interactions

To identify hydrogen bonds important for GF_2_/GF_3_ binding, hydrogen bond occupations of all systems were calculated as shown in Table 2, S2 and S3 Tables. In terms of GF_2_ binding, the N251A and N251Y mutations did not reduce the number of strong and medium hydrogen bonds between GF_2_ and binding residues. On the contrary, the total number of strong and medium hydrogen bonds of the mutant complexes is slightly more than that of the wild-type complex. These results suggest that these mutations may not significantly reduce the binding affinity of GF_2_, supporting the binding free energy results of GF_2_. In terms of GF_3_ binding, the N251A and N251Y mutations drastically reduce the number of strong and medium hydrogen bonds between GF_3_ and binding residues. These results suggest that these mutations probably cause the reduction of GF_3_ binding affinity, supporting the binding free energy results of GF_3_.

**Table 2:** Number of strong and medium hydrogen bonds formed between GF_2_/GF_3_ and binding residues in the GF_2_-LS_wt_, GF_2_-LS_N251A_, GF_2_-LS_N251Y_, GF_3_-LS_wt_, GF_3_-LS_N251A_, and GF_3_-LS_N251Y_ complexes.

Complex	Number of strong and medium hydrogen bond	Binding residues that form hydrogen bonds with GF_2_/GF_3_
**GF**_**2**_**-LS**_**wt**_	5 (4S*, 1M**)	Arg255, Glu349, Glu351
**GF**_**2**_**-LS**_**N251A**_	6 (4S, 2M)	fru-Asp93, Val123, Arg369, Arg442
**GF**_**2**_**-LS**_**N251Y**_	6 (4S, 2M)	fru-Asp93, Val123, Gln168, Tyr413, Arg442
**GF**_**3**_**-LS**_**wt**_	14 (9S, 5M)	Trp92, fru-Asp93, Thr126, Arg255, Glu349, Glu351, Arg369, Tyr438, Arg442
**GF**_**3**_**-LS**_**N251A**_	5 (3S, 2M)	Trp92, fru-Asp93, Glu351, Tyr413, Arg442
**GF**_**3**_**-LS**_**N251Y**_	4 (2S, 2M)	Trp92, fru-Asp93, Arg442

*S; Strong hydrogen bond

**M; medium hydrogen bond

**Fig 8:**
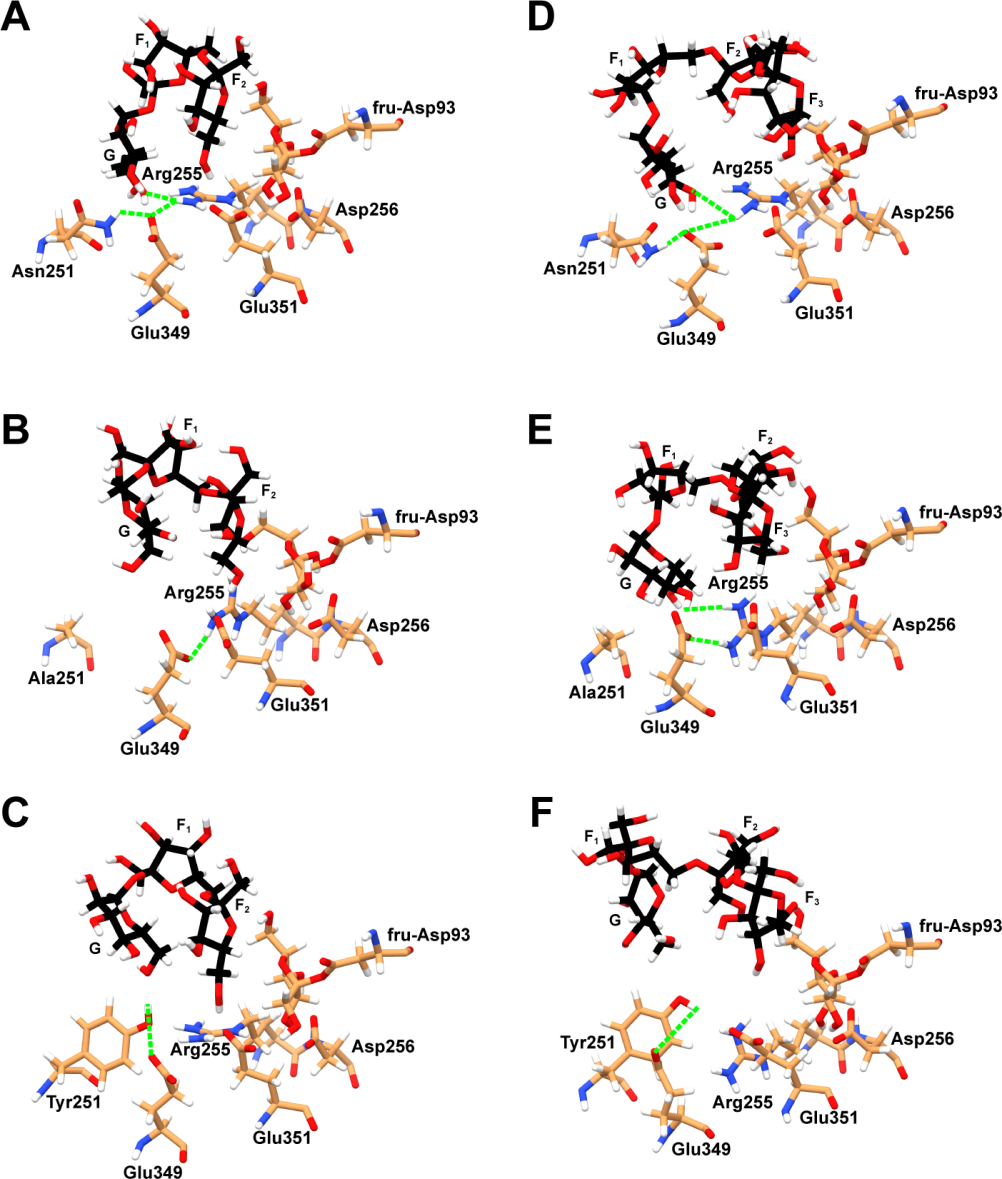
Hydrogen bond networks involving Arg255 and GF_2_/GF_3_ in A) GF_2_-LS_wt_, B) GF_2_-LS_N251A_, C) GF_2_-LS_N251Y_, D) GF_3_-LS_wt_, E) GF_3_-LS_N251A_ and F) GF_3_-LS_N251Y_ complexes at the beginning of the 80 ns MD simulations.

To determine the importance of Arg255 in GF_2_/GF_3_ binding in the active site of wild-type levansucrase, hydrogen bond networks involving Arg255 were identified as shown in Fig 8. At the beginning of the 80 ns MD simulations, GF_2_/GF_3_ formed hydrogen bond networks with Asn251, Glu349 and Arg255 in the wild-type complexes. The N251A and N251Y mutations disrupt these hydrogen bond networks in the mutant complexes; therefore, Arg255 could not effectively form hydrogen bonds with GF_2_/GF_3_ during the 80 ns MD simulations. However, there were other residues, instead of Asn251, Glu349 and Arg255, that later formed hydrogen bonds with GF_2_, still keeping it in a reasonable binding affinity and orientation for transfructosylation (Fig 3). These binding residues are fru-Asp93, Val123, Arg369, Arg442 for the N251A mutant, and fru-Asp93, Val123, Gln168, Tyr413 and Arg442 for the N251Y mutant (Table 2). These residues are different from the binding residues that formed hydrogen bonds with GF_2_ in the wild-type complex, and the total number of strong and medium hydrogen bonds formed between GF_2_ and the binding residues in the N251A and N251Y mutants is slightly more than that of the wild type. As a result, the binding conformations of GF_2_ in the active site of the mutants are slightly different from that of the wild type, but they are still in reasonable orientations and distances for transfructosylation. In terms of GF_3_ binding, the binding conformations of GF_3_ in the active sites of the N251A and N251Y mutants are drastically different from that of the wild type (Fig 3). Arg255 could not effectively form hydrogen bonds with GF_3_, and there are significantly less number of strong and medium hydrogen bonds formed between GF_3_ and the binding residues of the N251 A and N251Y mutants than that of the wild type (Table 2). Therefore, GF_3_ was not able to bind and stay in a favorable orientation for transfructosylation (Fig 3).

## Conclusions

In this work, MD was performed on the GF_2_-LS_wt_, GF_2_-LS_N251A_, GF_2_-LS_N251Y,_ GF_3_-LS_wt_, GF_3_-LS_N251A_ and GF_3_-LS_N251Y_ complexes to gain insight into the effects of N251A and N251Y mutations on the binding of GF_2_/GF_3_ in the active site of *Bacillus licheniformis* RN-01 levansucrase. Our results of binding free energies and hydrogen bond occupations as well as the distances between atoms necessary for transfructosylation of GF_3_-LS_wt_, GF_3_-LS_N251A_ and GF_3_-LS_N251Y_ complexes support the hypothesis that these mutations reduced GF_3_ binding affinity in active site of levansucrase with fructosyl-Asp93 intermediate and caused GF_3_ to be in an unfavorable orientation for transfructosylation; therefore, transfructosylation could not occur in GF_3_-LS_N251A_ and GF_3_-LS_N251Y_ complexes. As a result, only the wild type should be able to extend GF_3_ by one fructosyl residue to produce GF_4_, supporting the experimental results that the wild type can produce GF_4,_ but the N251A and N251Y mutants cannot effectively produce GF_4_. However, these mutations did not drastically change binding affinity or orientation of GF_2_ as shown by the binding free energy and hydrogen bond occupation results as well as the distances between atoms necessary for transfructosylation of GF_2_-LS_wt_, GF_2_-LS_N251A_ and GF_2_-LS_N251Y_ complexes. Therefore, the wild type, the N251A and N251Y mutants should be able to extend GF_2_ by one fructosyl residue to produce GF_3,_ supporting the experimental results that the wild type, the N251A and N251Y mutants can produce GF_3_. Moreover, the free energy decomposition results also suggest the importance of Arg255 in GF_2_/GF_3_ binding in the active site of the wild type. Our results also show that Arg255 formed hydrogen bond networks with GF_2_/GF_3_, Asn251 and Glu349 in the wild-type complexes at the beginning of the 80 ns MD simulations, and the N251A and N251Y mutations disrupted these hydrogen bond networks. Although these hydrogen bond networks were disrupted in the GF_2_-Ls_N251A_ and GF_2_-Ls_N251Y_ complexes, GF_2_ could still bind in a favorable orientation for transfructosylation in the active sites of these mutants probably because there were other residues binding and forming hydrogen bonds with GF_2_, and these interactions helped prevent misorientation of GF_2_. However, GF_3_ could not bind in a favorable orientation for transfructosylation in the active sites of these mutants because there was significantly less number of residues binding and forming hydrogen bonds with GF_3_ in the mutant complexes than that in the wild-type complex. Our study provides important and novel insight into the binding of GF_2_/ GF_3_ in the active site of *Bacillus licheniformis* RN-01 levansucrase and into how N251A and N251Y mutations may disrupt production of long-chain levan.

## Supporting information

S1 Fig Ramachandran plot of the homology model of *Bacillus licheniformis* RN-01 levansucrase. (TIF)

S2 Fig Superimposition of the crystal binding conformation (black) and best docked conformation (pink). (TIF)

S3 Fig The distance between O6 of the non-reducing end of GF_2_/GF_3_ and C2 of the fructosyl residue of fru-Asp93 during 0–80 ns: A) GF_2_-LS_wt_, B) GF_2_-LS_N251A_, C) GF_2_-LS_N251Y_, D) GF_3_-LS_wt_, E) GF_3_-LS_N251A_ and F) GF_3_-LS_N251Y_ complexes.
(TIF)

S4 Fig The multiple sequence alignment of *B*. *licheniformis* RN-01 levanscurase and levansucrase from Gram-positive bacteria such as *B*. *megaterium* [56], *B*. *amyloliquefaciens* [57], *B*. *atrophaeus* [58], *B*. *subtilis* [9], and *B*. *stearothermophilus* [59], and from Gram-negative bacteria such as *G*. *diazotrophicus* [60], *Z*. *mobilis* [11], *P*. *chlororaphis* [61], *R*. *aquatilis* [62] and *E*. *amylovora* [63]. (TIF)

S1 Table Structural alignment results of *Bacillus licheniformis* RN-01 levansucrase using PDBeFold. (DOCX)

S2 Table Hydrogen bond occupations of GF_2_-LS_wt_, GF_2_-LS_N251A_ and GF_2_-LS_N251Y_ complexes. (DOCX)

S3 Table Hydrogen bond occupations of GF_3_-LS_wt_, GF_3_-LS_N251A_ and GF_3_-LS_N251Y_ complexes. (DOCX)

## References

[1] Han J, Xu X, Gao C, Liu Z, Wu Z. Levan-producing *Leuconostoc citreum* strain BD1707 and its growth in tomato juice supplemented with sucrose. Applied and Environmental Microbiology. 2016;82(5):1383–90. 10.1128/AEM.02944-15 PMC4771333 26682858

[2] Srikanth R, Reddy CHS, Siddartha G, Ramaiah MJ, Uppuluri KB. Review on production, characterization and applications of microbial levan. Carbohydrate Polymers. 2015;120:102–14. 10.1016/j.carbpol.2014.12.003 25662693

[3] Han YW. Microbial levan. Advances in Applied Microbiology. 1990; 35: 171–94. 2205081 10.1016/s0065-2164(08)70244-2

[4] Arvidson SA, Rinehart BT, Gadala-Maria F. Concentration regimes of solutions of levan polysaccharide from *Bacillus* sp. Carbohydrate Polymers. 2006;65(2):144–9.

[5] Dal Bello F, Walter J, Hertel C, Hammes WP. *In vitro* study of prebiotic properties of levan-type exopolysaccharides from lactobacilli and non-digestible carbohydrates using denaturing gradient gel electrophoresis. Systematic and Applied Microbiology. 2001;24(2):232–7. 10.1078/0723-2020-00033 11518326

[6] Gibson GR, Probert HM, Van Loo J, Rastall RA, Roberfroid MB. Dietary modulation of the human colonic microbiota: updating the concept of prebiotics. Nutrition Research Reviews. 2004;17(2):259–75. 10.1079/NRR200479 19079930

[7] Kim KH, Chung CB, Kim YH, Kim KS, Han CS, Kim CH. Cosmeceutical properties of levan produced by *Zymomonas mobilis*. Journal of Cosmetic Science. 2005;56(6):395–406. 16538295

[8] Rairakhwada D, Pal A, Bhathena Z, Sahu N, Jha A, Mukherjee S. Dietary microbial levan enhances cellular non-specific immunity and survival of common carp (*Cyprinus carpio*) juveniles. Fish & Shellfish Immunology. 2007;22(5):477–86. 17158064 10.1016/j.fsi.2006.06.005

[9] Steinmetz M, Le Coq D, Aymerich S, Gonzy-Tréboul G, Gay P. The DNA sequence of the gene for the secreted *Bacillus subtilis* enzyme levansucrase and its genetic control sites. Molecular and General Genetics MGG. 1985;200(2):220–8. 2993818 10.1007/BF00425427

[10] Song KB, Seo JW, Kim MG, Rhee SK. Levansucrase of *Rahnella aquatilis* ATCC33071: gene cloning, expression, and levan formation. Annals of the New York Academy of Sciences. 1998;864(1):506–11. 9928133 10.1111/j.1749-6632.1998.tb10369.x

[11] Goldman D, Lavid N, Schwartz A, Shoham G, Danino D, Shoham Y. Two active forms of *Zymomonas mobilis* levansucrase an ordered microfibril structure of the enzyme promotes levan polymerization. Journal of Biological Chemistry. 2008;283(47):32209–17. 10.1074/jbc.M805985200 18809687

[12] Kang HK, Seo MY, Seo ES, Kim D, Chung SY, Kimura A, et al Cloning and expression of levansucrase from *Leuconostoc mesenteroides* B-512 FMC in *Escherichia coli*. Biochimica et Biophysica Acta (BBA)-Gene Structure and Expression. 2005;1727(1):5–15. 15652153 10.1016/j.bbaexp.2004.10.012

[13] Seibel J, Moraru R, Götze S, Buchholz K, Na’amnieh S, Pawlowski A, et al Synthesis of sucrose analogues and the mechanism of action of *Bacillus subtilis* fructosyltransferase (levansucrase). Carbohydrate Research. 2006;341(14):2335–49. 10.1016/j.carres.2006.07.001 16870166

[14] Nakapong S. Biochemical and structural characterization of levansucrase from *Bacillus licheniformis* RN-01. Ph.D. Thesis. Chulalongkorn University. 2011.

[15] Bryce R, Hillier I, Naismith J. Carbohydrate-protein recognition: molecular dynamics simulations and free energy analysis of oligosaccharide binding to concanavalin A. Biophysical Journal. 2001;81(3):1373–88. 10.1016/S0006-3495(01)75793-1 11509352 PMC1301617

[16] Cao R, Jin Y, Xu D. Recognition of cello-oligosaccharides by CBM17 from *Clostridium cellulovorans*: molecular dynamics simulation. The Journal of Physical Chemistry B. 2012;116(21):6087–96. 10.1021/jp3010647 22582874

[17] Fadda E, Woods RJ. Molecular simulations of carbohydrates and protein–carbohydrate interactions: motivation, issues and prospects. Drug Discovery Today. 2010;15(15–16):596–609. 10.1016/j.drudis.2010.06.001 20594934 PMC3936463

[18] Liu J-Y, Chen X-E, Zhang Y-L. Insights into the key interactions between human protein phosphatase 5 and cantharidin using molecular dynamics and site-directed mutagenesis bioassays. Scientific Reports. 2015;5:12359 10.1038/srep12359 26190207 PMC4507179

[19] Olarte-Avellaneda S, Rodríguez-López A, Patiño JD, Alméciga-Díaz CJ, Sánchez OF. In Silico Analysis of the Structure of Fungal Fructooligosaccharides-Synthesizing Enzymes. Interdisciplinary Sciences: Computational Life Sciences. 2016:1–15. 10.1007/s12539-016-0154-y 26879960

[20] Singh PK, Joseph J, Goyal S, Grover A, Shukla P. Functional analysis of the binding model of microbial inulinases using docking and molecular dynamics simulation. Journal of Molecular Modeling. 2016;22(4):69 10.1007/s00894-016-2935-y 26956120

[21] Karplus M, McCammon JA. Molecular dynamics simulations of biomolecules. Nature Structural and Molecular Biology. 2002;9(9):646. 10.1038/nsb0902-646 12198485

[22] Case D, Babin V, Berryman J, Betz R, Cai Q, Cerutti D, et al AMBER 14, 2014 University of California, San Francisco.

[23] Kirschner KN, Yongye AB, Tschampel SM, González‐Outeiriño J, Daniels CR, Foley BL, et al GLYCAM06: a generalizable biomolecular force field. Carbohydrates. Journal of Computational Chemistry. 2008;29(4):622–55. 10.1002/jcc.20820 17849372 PMC4423547

[24] Arnold K, Bordoli L, Kopp J, Schwede T. The SWISS-MODEL workspace: a web-based environment for protein structure homology modelling. Bioinformatics. 2006;22(2):195–201. 10.1093/bioinformatics/bti770 16301204

[25] Biasini M, Bienert S, Waterhouse A, Arnold K, Studer G, Schmidt T, et al SWISS-MODEL: modelling protein tertiary and quaternary structure using evolutionary information. Nucleic Acids Research. 2014;42(W1):W252–W8. 24782522 10.1093/nar/gku340 PMC4086089

[26] Guex N, Peitsch MC, Schwede T. Automated comparative protein structure modeling with SWISS‐MODEL and Swiss‐PdbViewer: A historical perspective. Electrophoresis. 2009;30(S1). 10.1002/elps.200900140 19517507

[27] Kiefer F, Arnold K, Künzli M, Bordoli L, Schwede T. The SWISS-MODEL Repository and associated resources. Nucleic Acids Research. 2008;37(suppl_1):D387–D92. 18931379 10.1093/nar/gkn750 PMC2686475

[28] Meng G, Fütterer K. Structural framework of fructosyl transfer in *Bacillus subtilis* levansucrase. Nature Structural and Molecular Biology. 2003;10(11):935. 10.1038/nsb974 14517548

[29] Lovell SC, Davis IW, Arendall WB III, De Bakker PI, Word JM, Prisant MG, et al Structure validation by Cα geometry: ϕ, ψ and Cβ deviation. Proteins: Structure, Function, and Bioinformatics. 2003;50(3):437–50. 10.1002/prot.10286 12557186

[30] Gordon JC, Myers JB, Folta T, Shoja V, Heath LS, Onufriev A. H++: a server for estimating p K as and adding missing hydrogens to macromolecules. Nucleic Acids Research. 2005;33(suppl_2):W368–W71. 15980491 10.1093/nar/gki464 PMC1160225

[31] Dennington R, Keith T, Millam J. GaussView, version 5, 2009. Semichem Inc, Shawnee Mission, KS.

[32] Frisch M, Trucks G, Schlegel HB, Scuseria G, Robb M, Cheeseman J, et al Gaussian 09 (Revision C. 01), Gaussian. Inc, Wallingford, CT 2010.

[33] Trott O, Olson AJ. AutoDock Vina: improving the speed and accuracy of docking with a new scoring function, efficient optimization, and multithreading. Journal of Computational Chemistry. 2010;31(2):455–61. 10.1002/jcc.21334 19499576 PMC3041641

[34] Feig M, Karanicolas J, Brooks CL III. MMTSB Tool Set: enhanced sampling and multiscale modeling methods for applications in structural biology. Journal of Molecular Graphics and Modelling. 2004;22(5):377–95. 10.1016/j.jmgm.2003.12.005 15099834

[35] AW, Williamson MJ, Xu D, Poole D, Le Grand S, Walker RC. Routine microsecond molecular dynamics simulations with AMBER on GPUs. 1. Generalized born. Journal of Chemical Theory and Computation. 2012;8(5):1542–55. 10.1021/ct200909j 22582031 PMC3348677

[36] Le Grand S, Götz AW, Walker RC. SPFP: Speed without compromise—A mixed precision model for GPU accelerated molecular dynamics simulations. Computer Physics Communications. 2013;184(2):374–80.

[37] Salomon-Ferrer R, Götz AW, Poole D, Le Grand S, Walker RC. Routine microsecond molecular dynamics simulations with AMBER on GPUs. 2. Explicit solvent particle mesh Ewald. Journal of Chemical Theory and Computation. 2013;9(9):3878–88. 10.1021/ct400314y 26592383

[38] York DM, Darden TA, Pedersen LG. The effect of long‐range electrostatic interactions in simulations of macromolecular crystals: A comparison of the Ewald and truncated list methods. The Journal of Chemical Physics. 1993;99(10):8345–8.

[39] Wu X, Brooks BR. Self-guided Langevin dynamics simulation method. Chemical Physics Letters. 2003;381(3–4):512–8.

[40] Miller BR III, McGee TD Jr, Swails JM, Homeyer N, Gohlke H, Roitberg AE. MMPBSA. py: an efficient program for end-state free energy calculations. Journal of Chemical Theory and Computation. 2012;8(9):3314–21. 10.1021/ct300418h 26605738

[41] Swanson JM, Henchman RH, McCammon JA. Revisiting free energy calculations: a theoretical connection to MM/PBSA and direct calculation of the association free energy. Biophysical Journal. 2004;86(1):67–74. 14695250 10.1016/S0006-3495(04)74084-9 PMC1303837

[42] Genheden S, Ryde U. The MM/PBSA and MM/GBSA methods to estimate ligand-binding affinities. Expert Opinion on Drug Discovery. 2015;10(5):449–61. 10.1517/17460441.2015.1032936 25835573 PMC4487606

[43] Hou T, Wang J, Li Y, Wang W. Assessing the performance of the MM/PBSA and MM/GBSA methods. 1. The accuracy of binding free energy calculations based on molecular dynamics simulations. Journal of Chemical Information and Modeling. 2010;51(1):69–82. 10.1021/ci100275a 21117705 PMC3029230

[44] Hou T, Wang J, Li Y, Wang W. Assessing the performance of the molecular mechanics/Poisson Boltzmann surface area and molecular mechanics/generalized Born surface area methods. II. The accuracy of ranking poses generated from docking. Journal of Computational Chemistry. 2011;32(5):866–77. 10.1002/jcc.21666 20949517 PMC3043139

[45] Mena-Ulecia K, Tiznado W, Caballero J. Study of the differential activity of thrombin inhibitors using docking, QSAR, molecular dynamics, and MM-GBSA. PloS One. 2015;10(11):e0142774 10.1371/journal.pone.0142774 26599107 PMC4657979

[46] Rastelli G, Rio AD, Degliesposti G, Sgobba M. Fast and accurate predictions of binding free energies using MM‐PBSA and MM‐GBSA. Journal of Computational Chemistry. 2010;31(4):797–810. 10.1002/jcc.21372 19569205

[47] Sun H, Li Y, Shen M, Tian S, Xu L, Pan P, et al Assessing the performance of MM/PBSA and MM/GBSA methods. 5. Improved docking performance using high solute dielectric constant MM/GBSA and MM/PBSA rescoring. Physical Chemistry Chemical Physics. 2014;16(40):22035–45. 10.1039/c4cp03179b 25205360

[48] Virtanen SI, Niinivehmas SP, Pentikäinen OT. Case-specific performance of MM-PBSA, MM-GBSA, and SIE in virtual screening. Journal of Molecular Graphics and Modelling. 2015;62:303–18. 10.1016/j.jmgm.2015.10.012 26550792

[49] Xu L, Sun H, Li Y, Wang J, Hou T. Assessing the performance of MM/PBSA and MM/GBSA methods. 3. The impact of force fields and ligand charge models. The Journal of Physical Chemistry B. 2013;117(28):8408–21. 10.1021/jp404160y 23789789

[50] Ylilauri M, Pentikäinen OT. MMGBSA as a tool to understand the binding affinities of filamin–peptide interactions. Journal of Chemical Information and Modeling. 2013;53(10):2626–33. 10.1021/ci4002475 23988151

[51] Gohlke H, Kiel C, Case DA. Insights into protein–protein binding by binding free energy calculation and free energy decomposition for the Ras–Raf and Ras–RalGDS complexes. Journal of Molecular Biology. 2003;330(4):891–913. 12850155 10.1016/s0022-2836(03)00610-7

[52] Hou T, Zhang W, Case DA, Wang W. Characterization of domain–peptide interaction interface: a case study on the amphiphysin-1 SH3 domain. Journal of Molecular Biology. 2008;376(4):1201–14. 10.1016/j.jmb.2007.12.054 18206907

[53] Niu Y, Pan D, Shi D, Bai Q, Liu H, Yao X. Influence of chirality of crizotinib on its MTH1 protein inhibitory activity: insight from molecular dynamics simulations and binding free energy calculations. PloS One. 2015;10(12):e0145219 10.1371/journal.pone.0145219 26677850 PMC4683072

[54] Zuo Z, Liu J. Cas9-catalyzed DNA cleavage generates staggered ends: Evidence from molecular dynamics simulations. Scientific reports. 2016;6:37584. 10.1038/srep37584 PMC5118739 27874072

[55] Wuerges J, Caputi L, Cianci M, Boivin S, Meijers R, Benini S. The crystal structure of *Erwinia amylovora* levansucrase provides a snapshot of the products of sucrose hydrolysis trapped into the active site. Journal of Structural Biology. 2015;191(3):290–8. 10.1016/j.jsb.2015.07.010 26208466

[56] Homann A, Biedendieck R, Götze S, Jahn D, Seibel J. Insights into polymer versus oligosaccharide synthesis: mutagenesis and mechanistic studies of a novel levansucrase from *Bacillus megaterium*. Biochemical Journal. 2007;407(2):189–98. 10.1042/BJ20070600 17608626 PMC2049016

[57] Rairakhwada D, Seo J-W, Seo M-y, Kwon O, Rhee S-K, Kim CH. Gene cloning, characterization, and heterologous expression of levansucrase from *Bacillus amyloliquefaciens*. Journal of Industrial Microbiology & Biotechnology. 2010;37(2):195–204. 19916084 10.1007/s10295-009-0664-2

[58] González-Garcinuño Á, Tabernero A, Sánchez-Álvarez JM, Galán MA, del Valle EMM. Effect of bacteria type and sucrose concentration on levan yield and its molecular weight. Microbial Cell Factories. 2017;16(1):91 10.1186/s12934-017-0703-z 28535808 PMC5442672

[59] Li Y, Triccas JA, Ferenci T. A novel levansucrase–levanase gene cluster in *Bacillus stearothermophilus* ATCC129801. Biochimica et Biophysica Acta (BBA)-Gene Structure and Expression. 1997;1353(3):203–8. 9349714 10.1016/s0167-4781(97)00103-6

[60] Martínez-Fleites C, Ortíz-Lombardía M, Pons T, Tarbouriech N, Taylor EJ, Arrieta JG, et al Crystal structure of levansucrase from the Gram-negative bacterium *Gluconacetobacter diazotrophicus*. Biochemical Journal. 2005;390(1):19–27. 15869470 10.1042/BJ20050324 PMC1188265

[61] Visnapuu T, Mardo K, Mosoarca C, Zamfir AD, Vigants A, Alamäe T. Levansucrases from *Pseudomonas syringae* pv. tomato and *P*. *chlororaphis* subsp. aurantiaca: Substrate specificity, polymerizing properties and usage of different acceptors for fructosylation. Journal of Biotechnology. 2011;155(3):338–49. 10.1016/j.jbiotec.2011.07.026 21820018

[62] Kim MG, Seo JW, Song K-B, Kim C-H, Chung BH, Rhee S-K. Levan and fructosyl derivatives formation by a recombinant levansucrase from *Rahnella aquatilis*. Biotechnology Letters. 1998;20(4):333–6.

[63] Geier G, Geider K. Characterization and influence on virulence of the levansucrase gene from the fireblight pathogen *Erwinia amylovora*. Physiological and Molecular Plant Pathology. 1993;42(6):387–404.

[64] Krissinel E, Henrick K. Secondary‐structure matching (SSM), a new tool for fast protein structure alignment in three dimensions. Acta Crystallographica Section D. 2004;60(12‐1):2256–68. 10.1107/S0907444904026460 15572779

[65] Strube CP, Homann A, Gamer M, Jahn D, Seibel J, Heinz DW. Polysaccharide synthesis of the levansucrase SacB from *Bacillus megaterium* is controlled by distinct surface motifs. Journal of Biological Chemistry. 2011:jbc. M110. 203166. 10.1074/jbc.M110.203166 PMC3093834 21454585

