# Supplementary figures and images for: Molecular dynamics provides insight into how N251A and N251Y mutations in the active site of Bacillus licheniformis RN-01 levansucrase disrupt production of long-chain levan
Source: PLoS One. 2018 Oct 2;13(10):e0204915. doi: 10.1371/journal.pone.0204915 (PMC6168164; doi:10.1371/journal.pone.0204915)

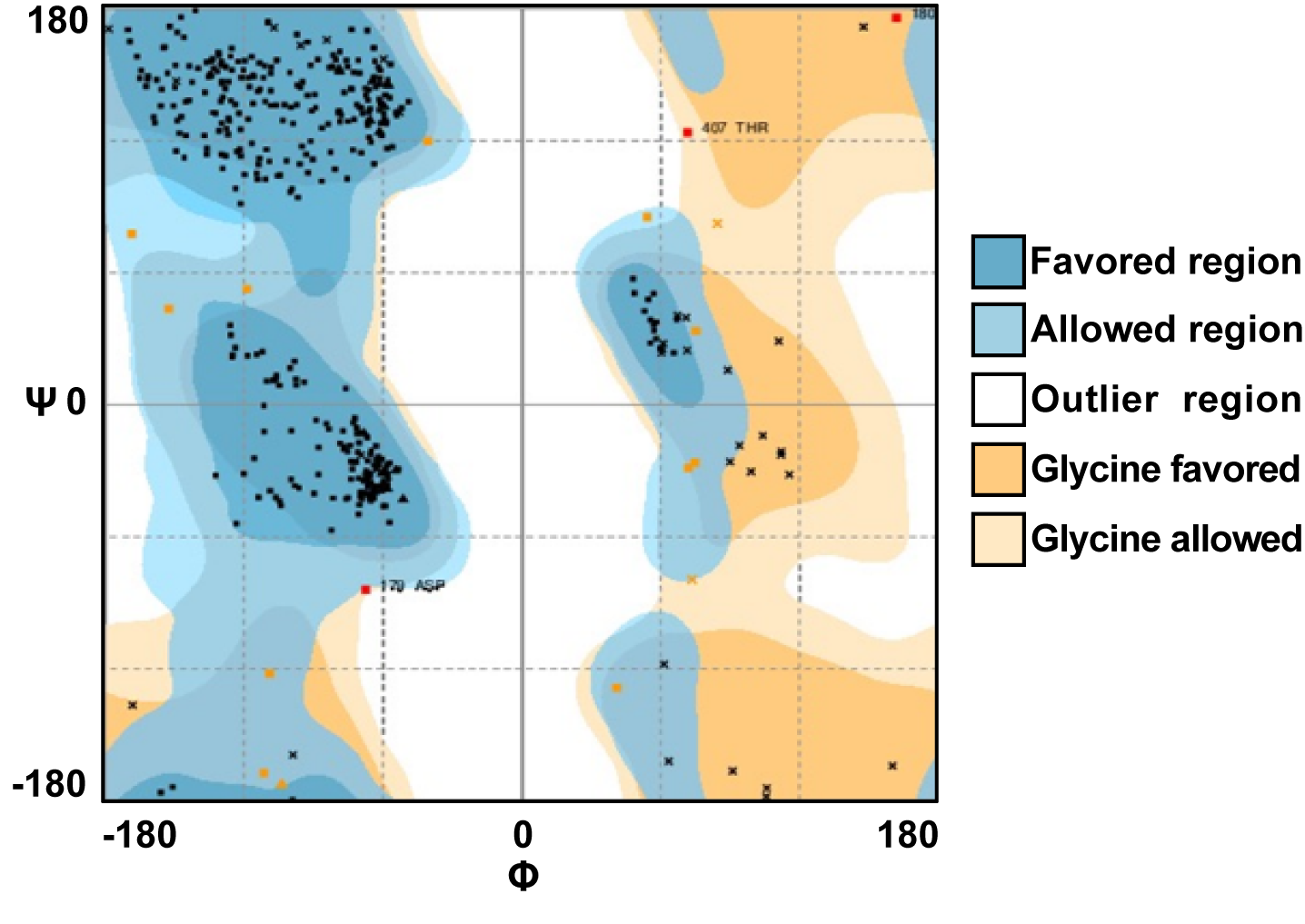

Supplement: S1 Fig — (TIF) [file pone.0204915.s001.tif]

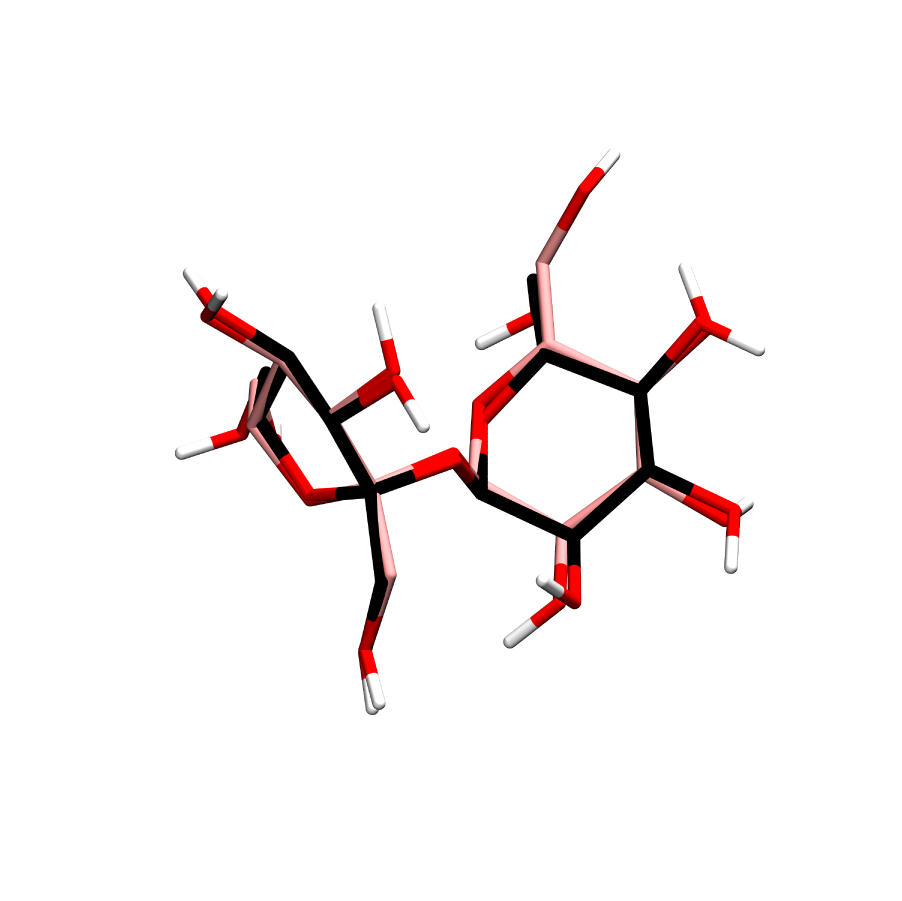

Supplement: S2 Fig — (TIF) [file pone.0204915.s002.tif]

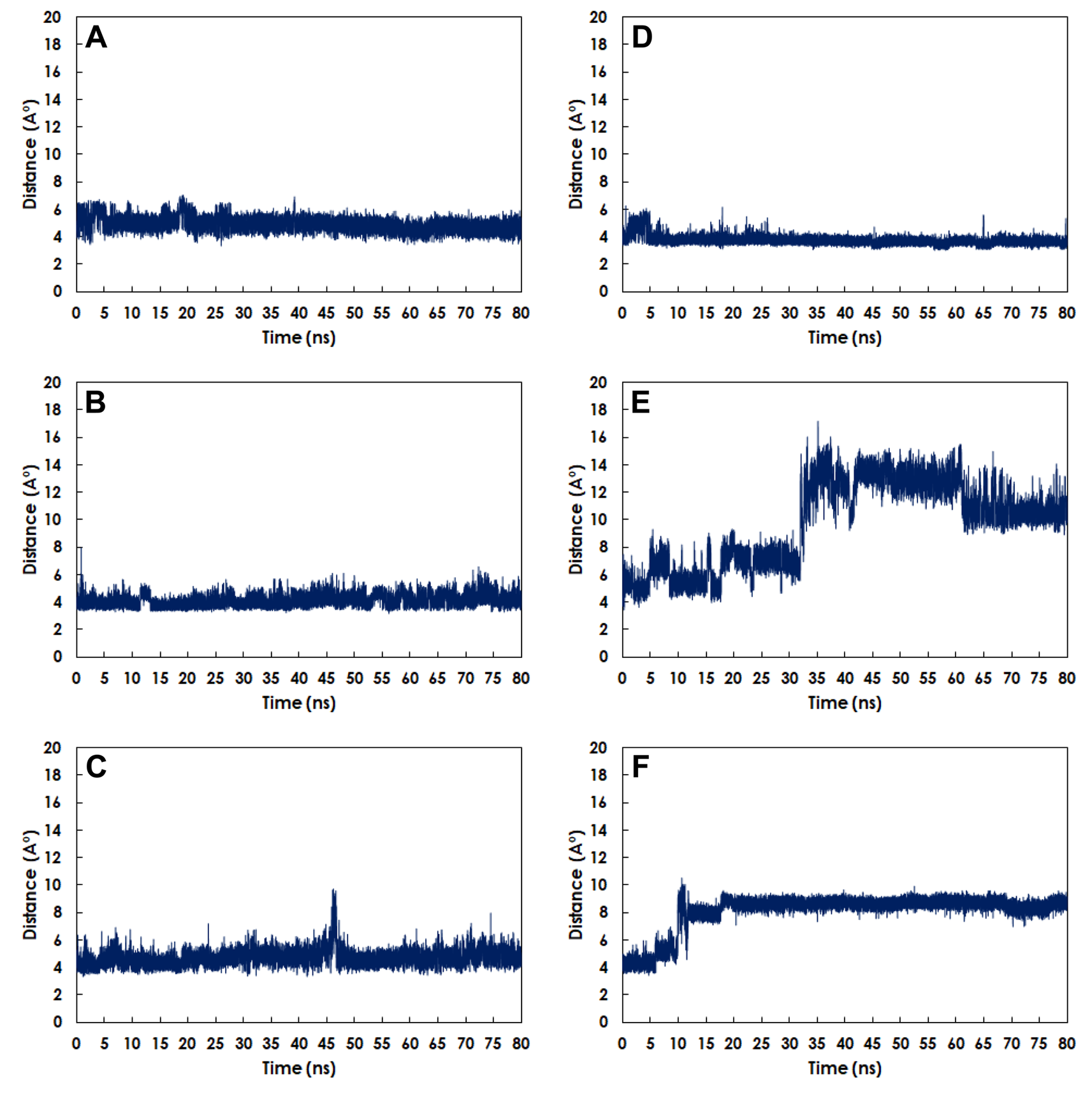

Supplement: S3 Fig — The distance between O6 of the non-reducing end of GF2/GF3 and C2 of the fructosyl residue of fru-Asp93 during 0–80 ns: A) GF2-LSwt, B) GF2-LSN251A, C) GF2-LSN251Y, D) GF3-LSwt, E) GF3-LSN251A and F) GF3-LSN251Y complexes. (TIF) [file pone.0204915.s003.tif]

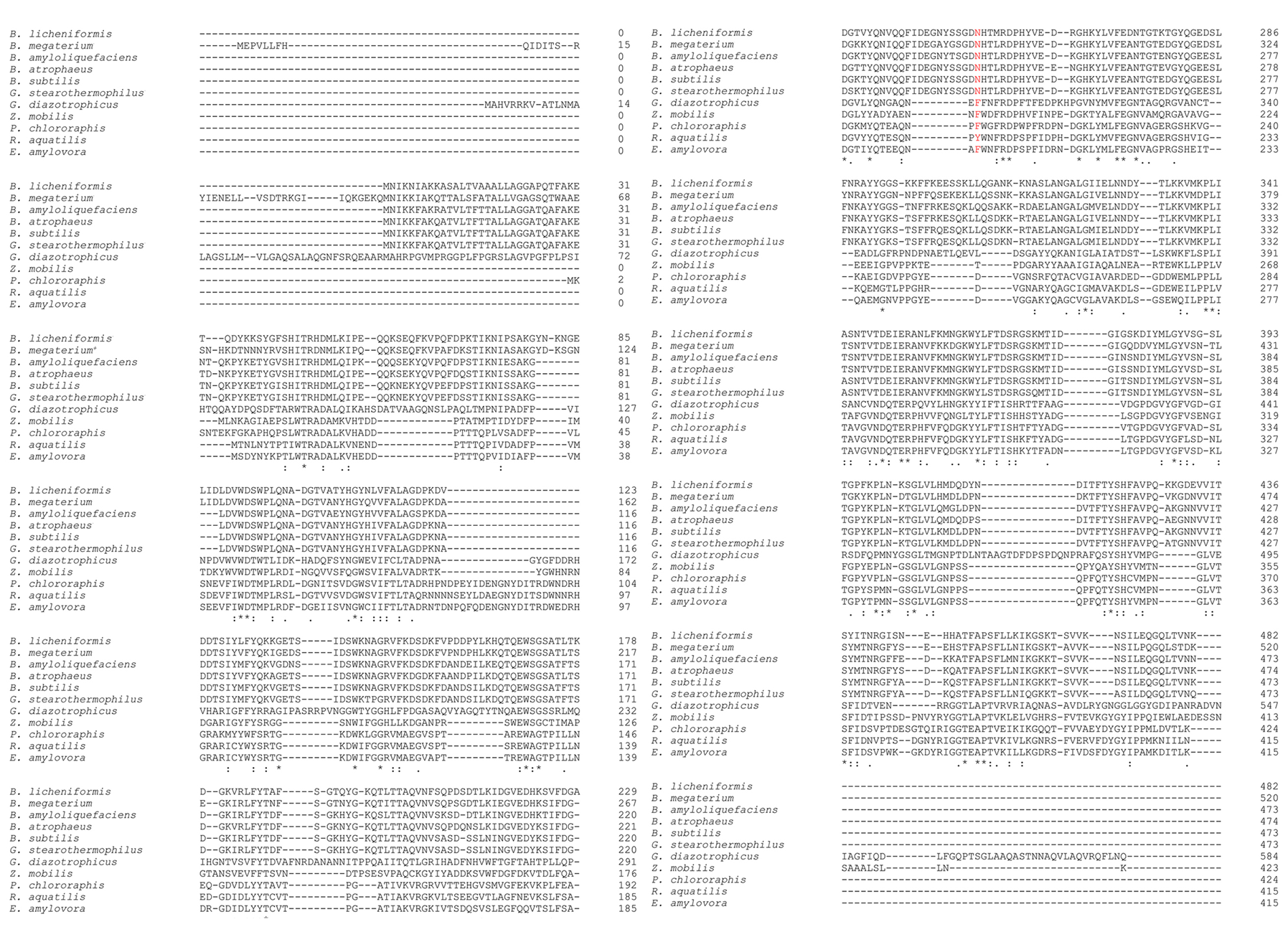

Supplement: S4 Fig — (TIF) [file pone.0204915.s004.tif]
