# Supplementary material for: Molecular dynamics provides insight into how N251A and N251Y mutations in the active site of Bacillus licheniformis RN-01 levansucrase disrupt production of long-chain levan
Source: PLoS One. 2018 Oct 2;13(10):e0204915. doi: 10.1371/journal.pone.0204915 (PMC6168164; doi:10.1371/journal.pone.0204915)
Supplement: S1 Table — (DOCX) [file pone.0204915.s005.docx]

**S1 Table.** Structural alignment results of *Bacillus licheniformis* RN-01 levansucrase using PDBeFold.

| **#** | **Scoring** | | | [**RMSD**](javascript:openWindow('/msd-srv/ssm/rl_rmsd.html',500,300);) | **N_align_** | [**N_g_**](javascript:openWindow('/msd-srv/ssm/rl_ngaps.html',500,300);) | **%_seq_** | **Query** | **Target (PDB entry)** | | | |
| --- | --- | --- | --- | --- | --- | --- | --- | --- | --- | --- | --- | --- |
|  | [**Q**](javascript:openWindow('/msd-srv/ssm/rl_qscore.html',500,300);) | [**P**](javascript:openWindow('/msd-srv/ssm/rl_zscore.html',500,300);) | [**Z**](javascript:openWindow('/msd-srv/ssm/rl_zscore.html',500,300);) |  |  |  |  | **%_sse_** | [**Match**](javascript:openWindow('/msd-srv/ssm/rl_match.html',500,300);) | **%_sse_** | **N_res_** | **Title** |
| [1](javascript:JSDetails(document.res_list_form,1)) | 0.95 | 49.3 | 21.0 | 0.19 | 434 | 4 | 77 | 77 | [1oyg:A](https://www.ebi.ac.uk/pdbe/entry/pdb/1oyg) | 77 | 440 | Crystal structure of *B. subtilis* levansucrase |
| [2](javascript:JSDetails(document.res_list_form,2)) | 0.95 | 49.0 | 21.0 | 0.21 | 434 | 4 | 77 | 77 | [3byj:A](https://www.ebi.ac.uk/pdbe/entry/pdb/3byj) | 77 | 440 | Crystal structure of *B. subtilis* levansucrase mutant D86A |
| [3](javascript:JSDetails(document.res_list_form,3)) | 0.95 | 49.0 | 21.0 | 0.22 | 434 | 4 | 77 | 81 | [3byk:A](https://www.ebi.ac.uk/pdbe/entry/pdb/3byk) | 81 | 440 | Crystal structure of *B. subtilis* levansucrase mutant D247A |
| [4](javascript:JSDetails(document.res_list_form,4)) | 0.95 | 47.3 | 20.6 | 0.26 | 434 | 4 | 77 | 77 | [3byl:A](https://www.ebi.ac.uk/pdbe/entry/pdb/3byl) | 77 | 440 | Crystal structure of *B. subtilis* levansucrase mutant E342A |
| [5](javascript:JSDetails(document.res_list_form,5)) | 0.95 | 48.8 | 20.9 | 0.27 | 434 | 4 | 77 | 77 | [1pt2:A](https://www.ebi.ac.uk/pdbe/entry/pdb/1pt2) | 77 | 440 | Crystal structure of levansucrase (E342A) complexed with sucrose |
| [6](javascript:JSDetails(document.res_list_form,6)) | 0.94 | 47.7 | 20.7 | 0.24 | 431 | 5 | 77 | 77 | [3byn:A](https://www.ebi.ac.uk/pdbe/entry/pdb/3byn) | 75 | 440 | Crystal structure of *B. subtilis* levansucrase mutant E342A bound to raffinose |
| [7](javascript:JSDetails(document.res_list_form,7)) | 0.88 | 41.2 | 19.2 | 0.66 | 430 | 5 | 75 | 77 | [3om2:A](https://www.ebi.ac.uk/pdbe/entry/pdb/3om2) | 75 | 448 | Crystal structure of *B. megaterium* levansucrase mutant D257A |
| [8](javascript:JSDetails(document.res_list_form,8)) | 0.85 | 41.1 | 19.2 | 0.72 | 424 | 7 | 75 | 77 | [3om7:C](https://www.ebi.ac.uk/pdbe/entry/pdb/3om7) | 75 | 448 | Crystal structure of *B. megaterium* levansucrase mutant Y247W |
| [9](javascript:JSDetails(document.res_list_form,9)) | 0.85 | 38.3 | 18.5 | 0.73 | 424 | 7 | 75 | 77 | [3om5:C](https://www.ebi.ac.uk/pdbe/entry/pdb/3om5) | 75 | 448 | Crystal structure of *B. megaterium* levansucrase mutant N252A |
| [10](javascript:JSDetails(document.res_list_form,10)) | 0.85 | 39.8 | 18.9 | 0.67 | 422 | 8 | 76 | 77 | [3om4:D](https://www.ebi.ac.uk/pdbe/entry/pdb/3om4) | 75 | 448 | Crystal structure of *B. megaterium* levansucrase mutant K373A |
| [11](javascript:JSDetails(document.res_list_form,11)) | 0.84 | 35.3 | 17.7 | 0.73 | 423 | 6 | 76 | 74 | [3om4:A](https://www.ebi.ac.uk/pdbe/entry/pdb/3om4) | 72 | 448 | Crystal structure of *B. megaterium* levansucrase mutant K373A |
| [12](javascript:JSDetails(document.res_list_form,12)) | 0.84 | 36.2 | 18.1 | 0.74 | 423 | 8 | 76 | 74 | [3om5:B](https://www.ebi.ac.uk/pdbe/entry/pdb/3om5) | 72 | 448 | Crystal structure of *B. megaterium* levansucrase mutant N252A |
| [13](javascript:JSDetails(document.res_list_form,13)) | 0.84 | 39.1 | 18.9 | 0.75 | 423 | 6 | 76 | 77 | [3om5:A](https://www.ebi.ac.uk/pdbe/entry/pdb/3om5) | 75 | 448 | Crystal structure of *B. megaterium* levansucrase mutant N252A |
| [14](javascript:JSDetails(document.res_list_form,14)) | 0.84 | 39.0 | 18.7 | 0.70 | 421 | 7 | 76 | 77 | [3om5:D](https://www.ebi.ac.uk/pdbe/entry/pdb/3om5) | 75 | 448 | Crystal structure of *B. megaterium* levansucrase mutant N252A |
| [15](javascript:JSDetails(document.res_list_form,15)) | 0.84 | 39.5 | 18.8 | 0.70 | 421 | 7 | 76 | 77 | [3om7:A](https://www.ebi.ac.uk/pdbe/entry/pdb/3om7) | 73 | 448 | Crystal structure of *B. megaterium* levansucrase mutant Y247W |
| [16](javascript:JSDetails(document.res_list_form,16)) | 0.84 | 39.7 | 19.0 | 0.74 | 422 | 7 | 76 | 77 | [3om4:B](https://www.ebi.ac.uk/pdbe/entry/pdb/3om4) | 75 | 448 | Crystal structure of *B. megaterium* levansucrase mutant K373A |
| [17](javascript:JSDetails(document.res_list_form,17)) | 0.84 | 38.7 | 18.9 | 0.75 | 422 | 7 | 76 | 77 | [3om7:D](https://www.ebi.ac.uk/pdbe/entry/pdb/3om7) | 73 | 448 | Crystal structure of *B. megaterium* levansucrase mutant Y247W |
| [18](javascript:JSDetails(document.res_list_form,18)) | 0.84 | 40.7 | 19.3 | 0.72 | 421 | 7 | 76 | 77 | [3om6:B](https://www.ebi.ac.uk/pdbe/entry/pdb/3om6) | 75 | 448 | Crystal structure of *B. megaterium* levansucrase mutant Y247A |
| [19](javascript:JSDetails(document.res_list_form,19)) | 0.83 | 41.9 | 19.6 | 0.74 | 421 | 7 | 76 | 77 | [3om7:B](https://www.ebi.ac.uk/pdbe/entry/pdb/3om7) | 73 | 448 | Crystal structure of *B. megaterium* levansucrase mutant Y247W |
| [20](javascript:JSDetails(document.res_list_form,20)) | 0.83 | 36.9 | 18.1 | 0.77 | 420 | 8 | 75 | 77 | [3om4:C](https://www.ebi.ac.uk/pdbe/entry/pdb/3om4) | 75 | 448 | Crystal structure of *B. megaterium* levansucrase mutant K373A |
