# Supplementary material for: Molecular dynamics provides insight into how N251A and N251Y mutations in the active site of Bacillus licheniformis RN-01 levansucrase disrupt production of long-chain levan
Source: PLoS One. 2018 Oct 2;13(10):e0204915. doi: 10.1371/journal.pone.0204915 (PMC6168164; doi:10.1371/journal.pone.0204915)
Supplement: S2 Table — (DOCX) [file pone.0204915.s006.docx]

**S2 Table.** Hydrogen bond occupations of GF_2_-LS_wt_, GF_2_-LS_N251A_ and GF_2_-LS_N251Y_ complexes.

| **Acceptor** | **DonorH** | **Hydrogen bond occupancy (%)** | | |
| --- | --- | --- | --- | --- |
|  |  | **GF_2_-LS_wt_** | **GF_2_-LS_N251A_** | **GF_2_-LS_N251Y_** |
| F_1_ of GF_2_@O1 | fru-Asp93@H16 | - | 51.8 | - |
| F_2_ of GF_2_@O5 | fru-Asp93@H16 | - | - | 82.8 |
| Val123@O | F_1_ of GF_2_@H4O | - | 86.7 | 96.0 |
| Val123@O | F_2_ of GF_2_@H1 | - | - | 58.2 |
| F_1_ of GF_2_@O4 | Gln168@HE21 | - | - | 79.8 |
| F_2_ of GF_2_@O6 | Arg255@HH12 | 99.4 | - | - |
| F_2_ of GF_2_@O6 | Arg255@HH22 | 59.4 | - | - |
| G of GF_2_@O3 | Glu349@H3O | 80.5 | - | - |
| G of GF_2_@O4 | Glu349@H4O | 79.2 | - | - |
| F_2_ of GF_2_@O6 | Glu351@H6O | 99.4 | - | - |
| F_2_ of GF_2_@O4 | Arg369@HH11 | 0.1 | 73.1 | - |
| F_2_ of GF_2_@O6 | Arg369@HH22 | 0.8 | 75.2 | 48.5 |
| Tyr413@OH | F_2_ of GF_2_@H4O | 0.2 | 0.2 | 53.4 |
| F_2_ of GF_2_@O1 | Arg442@H | - | 93.0 | - |
| F_2_ of GF_2_@O1 | Arg442@HH11 | - | - | 97.6 |
| F_1_ of GF_2_@O4 | Arg442@HH11 | 24 | 98.5 | - |
