# Supplementary material for: Molecular dynamics provides insight into how N251A and N251Y mutations in the active site of Bacillus licheniformis RN-01 levansucrase disrupt production of long-chain levan
Source: PLoS One. 2018 Oct 2;13(10):e0204915. doi: 10.1371/journal.pone.0204915 (PMC6168164; doi:10.1371/journal.pone.0204915)
Supplement: S3 Table — (DOCX) [file pone.0204915.s007.docx]

**S3 Table.** Hydrogen bond occupations of GF_3_-LS_wt_, GF_3_-LS_N251A_ and GF_3_-LS_N251Y_ complexes.

| **Acceptor** | **DonorH** | **Hydrogen bond occupancy (%) of system** | | |
| --- | --- | --- | --- | --- |
|  |  | **GF_3_-Ls_WT_** | **GF_3_-Ls_N251A_** | **GF_3_-Ls_N251Y_** |
| Trp92@NE1 | F_2_ of GF_3_@H3O | - | 63.3 | - |
| F_2_ of GF_3_@O4 | Trp92@HE1 | 93.2 | - | - |
| fru-Asp93@O2 | F_2_ of GF_3_@H1O | - | 82.2 | - |
| fru-Asp93@O2 | F_3_ of GF_3_@H4O | - | - | 89.2 |
| F_3_ of GF_3_@O6 | fru-Asp93@H13 | 87.4 | - | - |
| fru-Asp93@O8 | F_3_ of GF_3_@H16 | - | - | 60.0 |
| Thr126@OG1 | F_2_ of GF_3_@H4O | 96.6 | - | - |
| G of GF_3_@O3 | Arg255@HH12 | 97.9 | 0.2 | - |
| Glu349@OE1 | G of GF_3_@H3O | 71.7 | 2.9 | - |
| Glu349@OE1 | G of GF_3_@H4O | 71.4 | 0.8 | - |
| Glu349@OE2 | G of GF_3_@H3O | 83.7 | 2.8 | - |
| Glu349@OE2 | G of GF_3_@H4O | 59.6 | 0.1 | - |
| Glu351@OE1 | G of GF_3_@H6O | 0.4 | 80.9 | - |
| Glu351@OE2 | G of GF_3_@H6O | 60.3 | 42.5 | - |
| F_3_ of GF_3_@O4 | Arg369@HH11 | 87.6 | - | - |
| F_3_ of GF_3_@O4 | Arg369@HH22 | 97.1 | - | 0.1 |
| Tyr413@OH | F_3_ of GF_3_@H4O | - | 62.2 | - |
| Tyr438@OH | F_3_ of GF_3_@H4O | 87.7 | 3.8 | 3.8 |
| F_2_ of GF_3_@O4 | Arg442@H | - | 95.8 | - |
| F_3_ of GF_3_@O1 | Arg442@H | 95.3 | - | - |
| F_3_ of GF_3_@O6 | Arg442@H | - | - | 98.4 |
| F_2_ of GF_3_@O3 | Arg442@HH12 | 55.7 | 0.3 | - |
| F_2_ of GF_3_@O4 | Arg442@HH11 | 0.1 | - | 58.7 |
